# Impact of congenital uterine anomalies on obstetric and perinatal outcomes: systematic review and meta-analysis

**DOI:** 10.52054/FVVO.16.1.004

**Published:** 2024-03-28

**Authors:** M Caballero Campo, F Pérez Milán, M Carrera Roig, E Moratalla Bartolomé, J.A. Domínguez Arroyo, J.L. Alcázar Zambrano, L Alonso Pacheco, J Carugno

**Affiliations:** Reproductive Medicine Unit, Obstetrics & Gynecology Department, Hospital General Universitario Gregorio Marañón, Madrid, Spain; Gregorio Marañón Institute for Health Research, Madrid, Spain; Universidad Complutense, Madrid, Spain; Reproductive Medicine Unit, Obstetrics & Gynecology Department, Hospital Universitario Doce de Octubre, Madrid, Spain; Gynecological Surgery and Endoscopy Unit, Hospital Universitario Ramón y Cajal, Madrid, Spain; Centro Extremeño de Reproducción Asistida, Badajoz, Spain; Universidad de Extremadura, Badajoz, Spain; Clínica Universidad de Navarra, Pamplona, Spain; Universidad de Navarra, Pamplona Spain; Gynecological Endoscopy Unit, Gutenberg Center, Xanit International Hospital, Málaga, Spain; Miller School of Medicine, Department of Obstetrics and Gynecology, University of Miami, Miami, FL, USA

**Keywords:** Congenital uterine anomalies, Müllerian anomalies, pregnancy outcome, obstetric complications, labour complications, neonatal outcome

## Abstract

**Background:**

Congenital uterine anomalies (CUA) can be associated with impairments of early and late pregnancy events.

**Objective:**

To assess the impact of CUA on reproductive outcomes in pregnancies conceived spontaneously or after assisted reproduction.

**Material and Methods:**

Systematic review and meta-analysis of cohort studies comparing patients with CUA versus women with normal uterus. A structured literature search was performed in leading scientific databases to identify prospective and retrospective studies. The Newcastle-Ottawa scale, adapted to AHRQ standards, was used to assess the risk of bias. Pooled odds ratios (OR) were calculated. Publication bias and statistical heterogeneity were assessed, and meta-regression was used to analyse the heterogeneity.

**Main outcome measures:**

Miscarriage, ectopic pregnancy, placental abruption, term, and premature rupture of membranes (PROM), malpresentation at delivery, preterm delivery prior to 37, 34 and 32 weeks, caesarean delivery, intrauterine growth restriction/small for gestational age, foetal mortality and perinatal mortality.

**Results:**

32 studies were included. CUAs increased significantly the risk of first/second trimester miscarriage (OR:1.54;95%CI:1.14-2.07), placental abruption (OR:5.04;3.60-7.04), PROM (OR:1.71;1.34-2.18), foetal malpresentation at delivery (OR:21.04;10.95-40.44), preterm birth (adjusted OR:4.34;3.59-5.21), a caesarean delivery (adjusted OR:7.69;4.17-14.29), intrauterine growth restriction/small for gestational age (adjusted OR:50;6.11-424), foetal mortality (OR:2.07;1.56-2.73) and perinatal mortality (OR:3.28;2.01-5.36).

**Conclusions:**

CUA increases the risk of complications during pregnancy, delivery, and postpartum. Complications most frequent in CUA patients were preterm delivery, foetal malpresentation, and caesarean delivery.

**What is new?:**

Bicornuate uterus was associated with the highest number of adverse outcomes, followed by didelphys, subseptate and septate uterus.

## Introduction

Congenital uterine anomalies (CUA) are uncommon entities caused by abnormal development, fusion, or resorption of Müllerian ducts during organogenesis, which results in defects in canalisation, unification, or conformation of Müllerian-derived structures. Their prevalence is difficult to assess, due to the lack of a universally accepted classification and differences in diagnostic methods or the population profiles reported in the available studies. A systematic review published by Chan et al. (2011b) estimated that CUA are present in 5.5% of infertile patients, in 13.3% of women with previous miscarriage and in 24.5% of women affected by both conditions.

CUA comprises a broad spectrum of congenital defects, characterised by different degrees of distortion of the uterine anatomy, which could generate different levels of perinatal risk. The association with reproductive outcomes has been extensively analysed. Until now, four systematic reviews on the association between CUA and obstetrical and perinatal risks have been published (Chan et al., 2011a; Grimbizis et al., 2001; Kim et al., 2021; Venetis et al., 2014). However, they include observational studies which are affected considerably by the risk of bias. Recently, several studies looking at the correlation between CUA and adverse pregnancy outcomes have been published.

The objective of this systematic review with meta-analysis is to evaluate the association between CUA with adverse pregnancy outcomes. Eligibility of studies will follow the actual criteria for risk of bias assessment of observational studies that complement score-based scales with domain-based evaluation, particularly concerning comparability between exposed and non-exposed patients.

## Materials and methods

### Protocol registration

The systematic review and meta-analysis protocol were defined according to MOOSE guidelines and registered at PROSPERO (CRD42023380794).

### Study selection

For eligibility, the following inclusion criteria were applied: a) Prospective and retrospective cohort studies analysing the effects of CUA on obstetrical and perinatal outcomes in spontaneous or ART pregnancies in patients affected by infertility, recurrent pregnancy loss, or general population; b) Fair or high quality studies with an adequate level of comparability between exposed and non-exposed patients; c) Peer-reviewed articles published in English, French, German or Spanish between January 1980 and April 2022.

As exclusion criteria we considered case-control design, insufficient information on the population considered or on diagnostic techniques used, lack of adjustment for potential confounders, studies considering comparisons other than those of interest, and non-comparative studies.

### Information sources and searches

A systematic review of primary studies was carried out by investigators in well-recognised scientific databases (MEDLINE, EMBASE, Current Contents, Web of Science, and Cochrane Database Register for Clinical Trials, ClinicalTrials.gov and Google Scholar). Search terms and limits are provided in Appendix 1 and were adapted to specific syntax in the different databases. Cross references were hand-searched.

### Studies selection and individual risk of bias assessment

Identified studies were initially classified according to title and abstract by two different authors. Studies concordantly selected were full text evaluated, and discrepancies were solved by consensus with a third evaluator.

Eligibility criteria were applied to the studies selected for full-text evaluation. Risk of bias was assessed using Newcastle-Ottawa Scale (NOS) for cohort studies, and qualified according to the standards of United States Agency for Healthcare Research and Quality (AHRQ) (Wells et al., 2021). Only studies qualified as good or fair quality (granted with 1 or 2 stars in the comparability domain), were included, considering age and parity as main potential confounders for all outcomes analysis. Reasons for exclusion were discussed and summarised. The studies selection process was in accordance with MOOSE Statement recommendations for systematic reviews (Stroup et al., 2000).

### Data collection, outcomes, and summary measures

Data extraction from selected studies was performed by one of the authors and verified by a co-author, using a pre-designed form. Definitions and classification categories for CUA used by authors were specifically checked.

We considered as outcomes first and second trimester miscarriage, ectopic pregnancy, placental abruption, term, and preterm premature rupture of membranes (PROM/PPROM) foetal malposition or abnormal presentation at delivery, preterm delivery, preterm delivery prior to 34 and prior to 32 weeks, caesarean delivery, intrauterine growth restriction or small for gestational age (IUGR/ SGA), foetal mortality and perinatal mortality. Odds ratios of these outcomes were considered as summary measures.

### Statistical analysis

Articles favourable for quantitative synthesis were meta-analysed applying a random-effects model (DerSimonian and Kacker, 2007). Pooled odds ratios (OR) and its 95% confidence interval were used as pooled effect measures.

Statistic heterogeneity was estimated by Cochran’s Q and I2 statistics. Q statistics with p values <0.05 was considered statistically significant. I2 values were evaluated considering critical thresholds previously defined (Borenstein et al., 2021; Higgins and Thompson, 2002).

Publication bias was assessed through funnel plots for each outcome. In case of obtaining non- conclusive plots, we applied Begg’s and Egger’s tests. Meta-regression based on random-effects models (Borenstein et al., 2021) was applied to adjust the effect on overall estimates affected by high statistical heterogeneity (I2 >50%), considering as covariates the type of cohort-study design (classical versus matched controls cohort studies), studied population (general population, infertile patients and previous pregnancy loss history) and type of pregnancy (singleton or multiple).

Review Manager 5.4.1 was used to calculate pooled estimated effects and heterogeneity and Stata software 17 was used for publication-bias and meta-regression analysis.

## Results

### Systematic review

**Figure 1:**
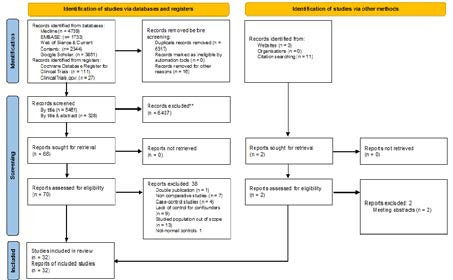
PRISMA flowchart diagram.

The structured searches identified 12794 reports. From these, 6461 were screened by title and 328 by title and abstract. A final subset of 78 studies were full-text evaluated (Figure 1), of which 32 (Ban-Frangez et al., 2009; Ben-Rafael et al., 1991; Cahen-Peretz et al., 2017; Cai et al., 2021; Cooney et al., 1998; Crane et al., 2012; Chen et al., 2018; Chen et al., 2019; Erez et al., 2007; Hiersch et al., 2016; Hua et al., 2011; Jayaprakasan et al., 2011; Kong et al., 2021; Leible et al., 1998; Li et al., 2017; Lu et al., 2021; Marianna et al., 2022; Mastrolia et al., 2017; Mastrolia et al., 2018; Ouyang et al., 2020; Ozgur et al., 2017; Pleş et al., 2018; Prior et al., 2018; Qiu et al., 2022; Saravelos et al., 2010; Sugiura-Ogasawara et al., 2010; Surrey et al., 2018; Takami et al., 2014; Tomaževič et al., 2010; Zambrotta et al., 2021; Zlopasa et al., 2007) fulfilled inclusion criteria (Table SI). Scores granted by Newcastle-Ottawa Score and quality assessment using AHRQ standards are presented in Appendix 2. Reasons for exclusion of non-included studies (Acién, 1993; Acién et al., 2014; Airoldi et al., 2005; Akar et al., 2005; Alonso Pacheco et al., 2019; Ben-Rafael et al., 1990; Colacurci et al., 1996; Chen et al., 2013; Elsokkary et al., 2018; Fedele and Bianchi, 1995; Fox et al., 2019; Fox et al., 2014; Gabbai et al., 2018; Ghi et al., 2012; Grimbizis et al., 2001; Hynes et al., 2021; Jaslow and Kutteh, 2013; Lavergne et al., 1996; Liang and Hu, 2010; Ludwin, 2018; Maneschi et al., 1995; Neal et al., 2019; Portuondo et al., 1986; Raga et al., 1997; Ravasia et al., 1999; Ridout et al., 2019; Rogers and Needham, 1985; Salim et al., 2003; Sendag et al., 2010; Shuiqing et al., 2002; Sorensen and Trauelsen, 1987; Sugiura-Ogasawara et al., 2015; Tofoski and Antovska, 2014; Tomazevic et al., 2007; Tonguc et al., 2011; Woelfer et al., 2001; Zhang et al., 2010; Zupi et al., 1996) are described in Table SII.

### Meta-analysis

#### Miscarriage

**Figure 2:**
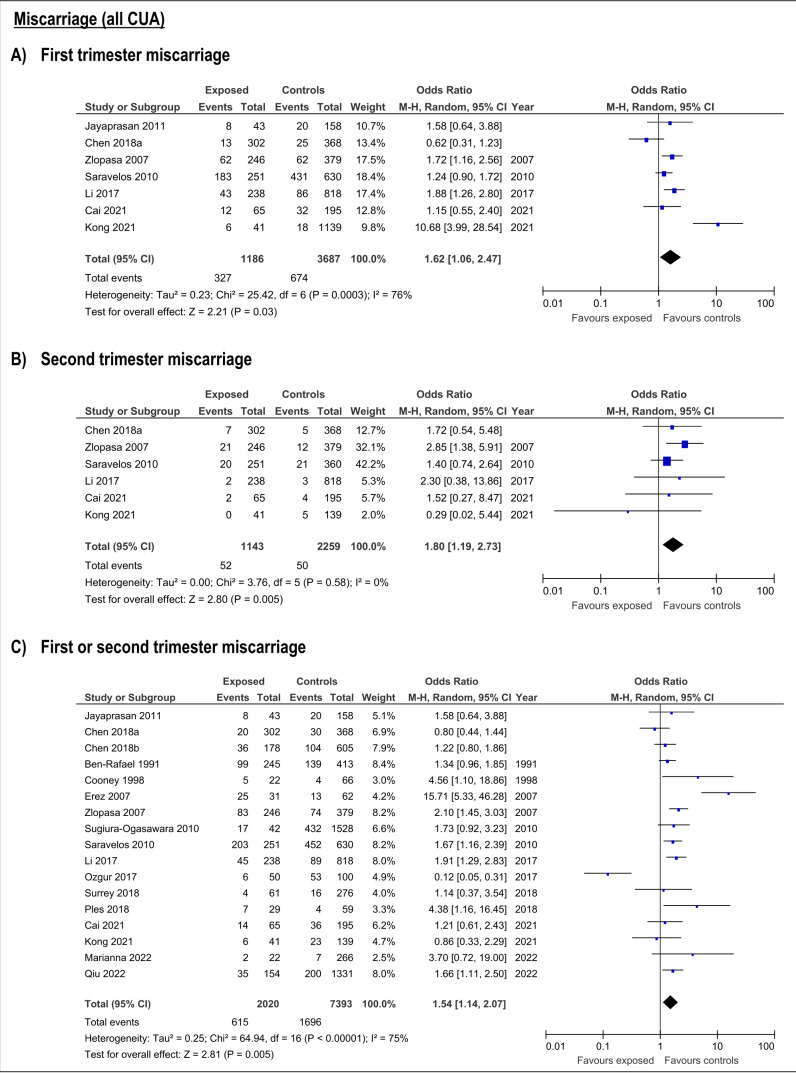
Forest plots of individual and pooled effects of CUA (combined) on first (A), second trimester (B) and any trimester (C) miscarriage risk.

**Figure 3:**
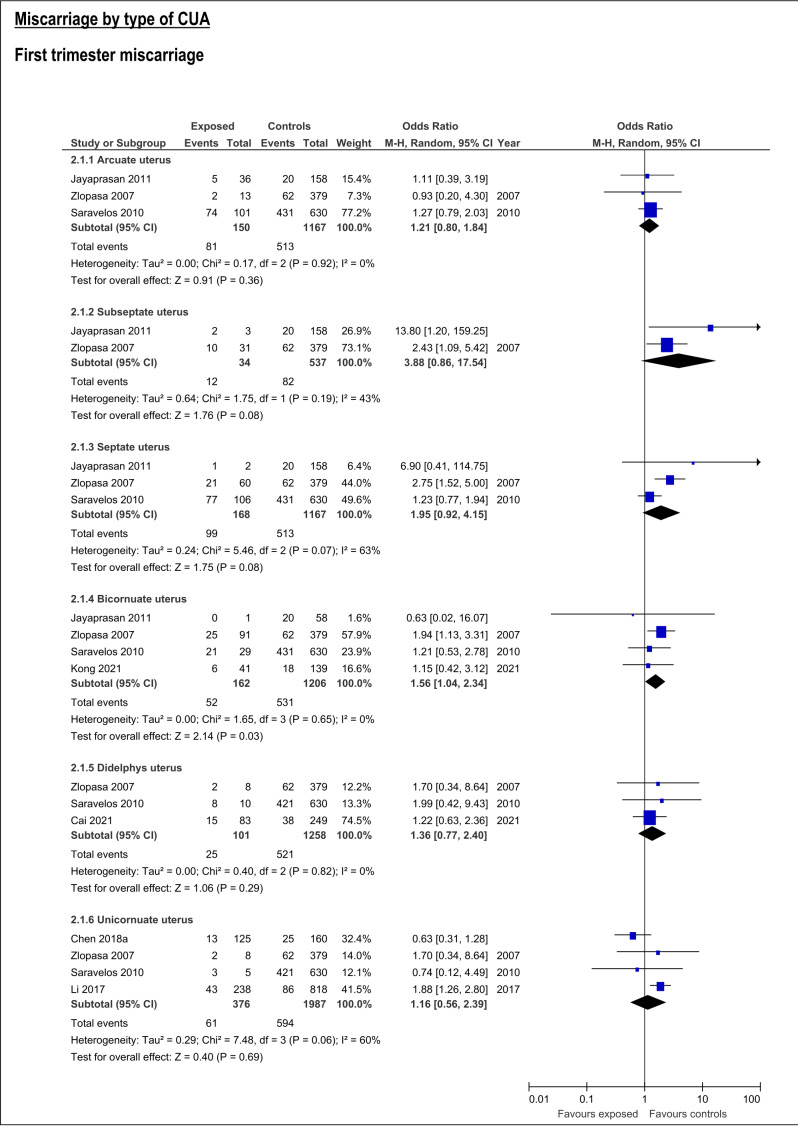
Forest plots of individual and pooled effects on first trimester miscarriage by type of CUA.

CUA increased the risk of first trimester miscarriage (OR:1.62, 95%CI:1.06-2.47; 7 studies; I2:76%) (Table SIII; Figure 2. This risk was only detectable for bicornuate uterus (OR:1.56; 95%CI:1.04-2.34, 4 studies; I2:0%), and resulted not significant for arcuate, septate, subseptate, didelphys and unicornuate uterus (Table SIII; Figure 3).

**Figure 4:**
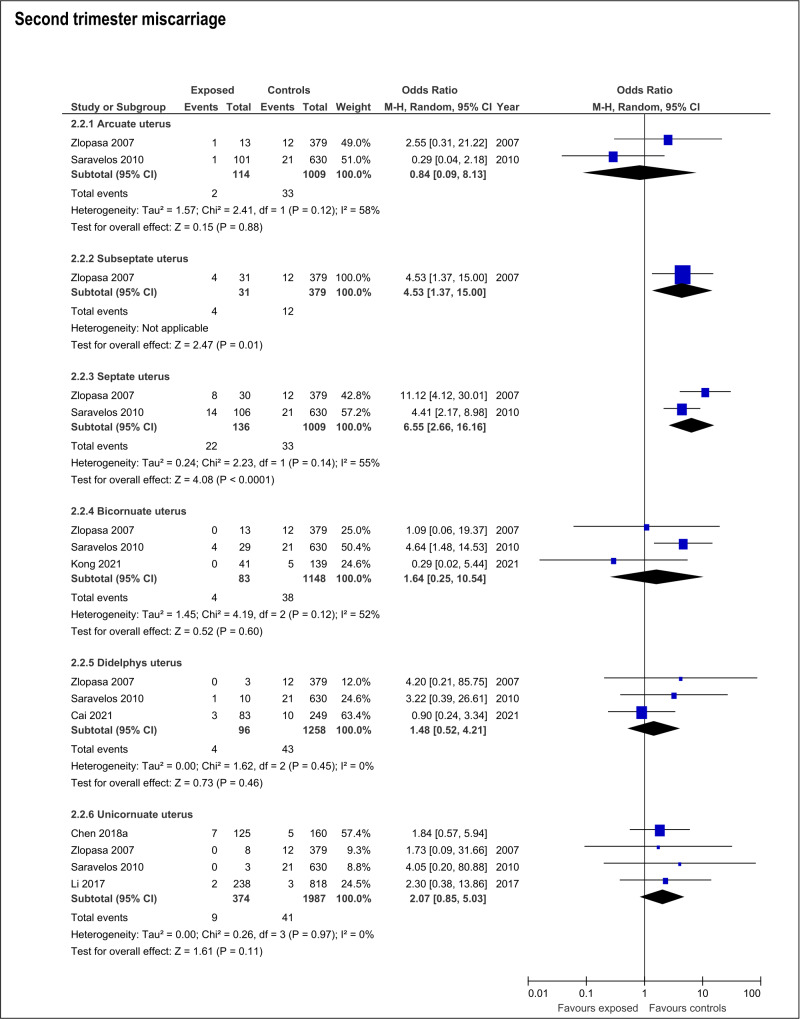
Forest plots of individual and pooled effects on second trimester miscarriage by type of CUA.

The presence of any CUA increased risk of second trimester miscarriage (OR:1.8; 95%CI:1.19- 2.73; 6 studies; I2:0%) (Table SIII; Figure 2). This risk was only present with septate uteri (OR:6.65; 95%CI:2.66-16.16; 2 studies; I2:55%). The estimated effect of the subseptate uterus on this outcome derived from a single study (OR:4.53; 95%CI:1.37-15.0) (Zlopasa et al., 2007). The rest of the evaluated anomalies (arcuate, didelphys, bicornuate and unicornuate uterus) showed no association with risk of second trimester miscarriage (Table SIII; Figure 4).

**Figure 5:**
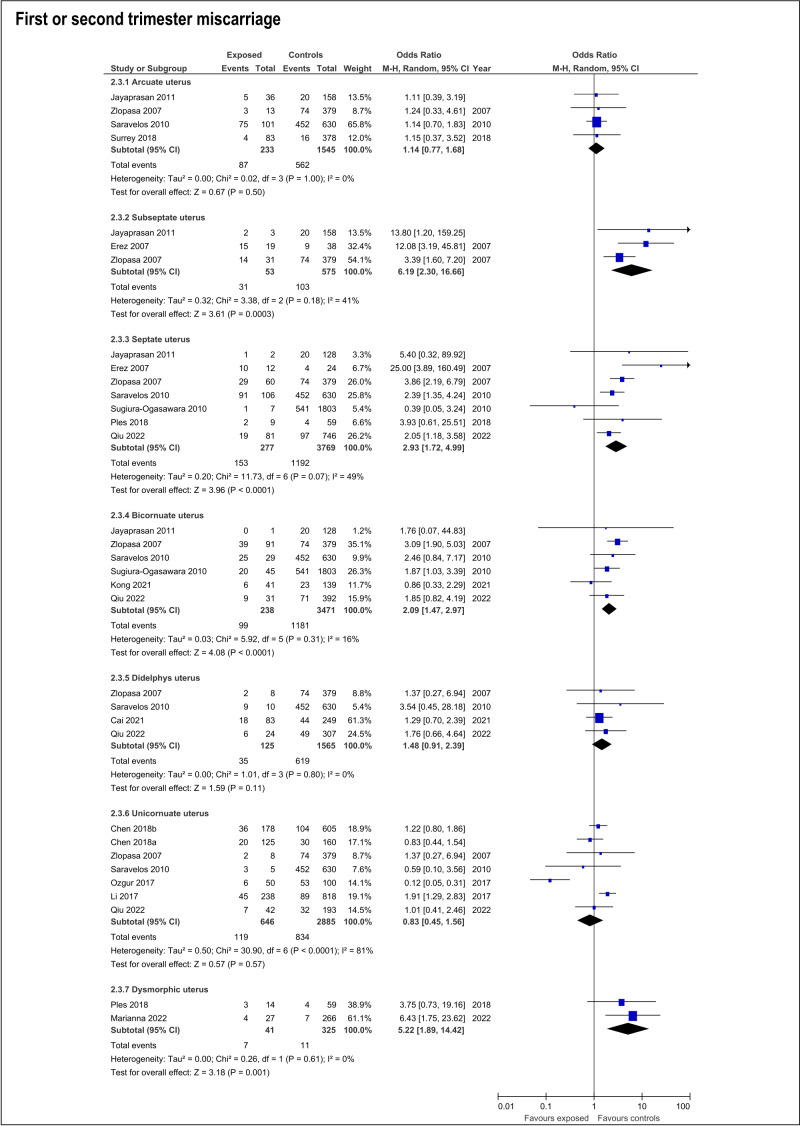
Forest plots of individual and pooled effects on any trimester miscarriage by type of CUA.

The risk of miscarriage in any trimester increases in the presence of any CUA (OR:1.54; 95%CI:1.14- 2.07; 17 studies; I2: 75%) (Table SIII; ). The specific anomalies that have increased risk of miscarriage in the first or second trimester were subseptate uterus (OR:6.19; 95%CI:2.3-16.66; 3 studies; I2:41%), septate (OR:2.93; 95%CI:1.72-4.99; 7 studies; I2:49%), bicornuate (OR:2.09; 95%CI:1.47-2.97; 6 studies; I2:16%) and T-shaped uterus (OR:5.22; 95%CI:1.89-14.42). On the contrary, arcuate, didelphys and unicornuate uterus did not increase the risk of miscarriage (Table SIII; Figure 5).

#### Ectopic pregnancy

Ectopic pregnancy is not increased in patients with CUA (OR:1.30; 95%CI:0.82-2.05; 6 studies; I2:0%) (Table SIII; Figure S1). When analysed by type of anomaly, only septate uteri had a significant increased risk of ectopic pregnancy (OR:2.04; 95%CI:2.03-4.04, 2 studies; I2:49%) (Qiu et al., 2022; Saravelos et al., 2010). Effects of arcuate, and T-shaped uterus were estimated each one from a single study (Marianna et al., 2022; Saravelos et al., 2010), whereas effects of didelphys, bicornuate and unicornuate uterus derived from the data of two (Qiu et al., 2022; Saravelos et al., 2010), three (Kong et al., 2021; Qiu et al., 2022; Saravelos et al., 2010) and four studies (Chen X. et al., 2018; Li et al., 2017; Qiu et al., 2022; Saravelos et al., 2010) respectively (Table SIII; Figure S2).

#### Placental abruption

Presence of any CUA increased risk of placental abruption (OR:5.04; 95%CI:3.66-7.04; 6 studies; I2:40%) (Table SIII; Figure S3). This event is more frequent in patients with subseptate (OR:17.45; 95%CI:5.05-60.22; 1 study) and bicornuate uterus (OR:12.11; 95%CI:3.14-46.74; 2 studies; I2:81%). No effects of septate, didelphys and unicornuate uterus were found analysing data from a single study (Takami et al., 2014) (Table SIII; Figure S4).

#### PROM/PPROM

Combined PROM-PPROM risk was increased in patients with CUA (OR:1.71; 95%CI:1.34-2.18; 9 studies; I2:65%) (Table SIII; Figure S5), specifically with bicornuate uterus (OR:1.79; 95%CI: 1.37- 2.33; 2 studies; I2: 0%). No effect was detected for unicornuate uterus (OR:0.46; 95%CI:0.18-1.21, 1 study) (Lu et al., 2021) (Table SIII; Figure S6).

#### Fetal Malpresentation at delivery

Fetal malpresentation at the time of delivery was consistently increased in women with CUA (OR:21.04; 95%CI:10.95-40.44; 7 studies; I2:97%) (Table SIII; Figure S7). Arcuate uterus (OR:11.38; 95%CI:1.49-87.07; 2 studies; I2:41%), subseptate uterus (OR:25.62; 95%CI:10.69-60.85; 2 studies; I2:25%), septate uterus (OR:45.48; 95%CI:16.97- 121.89; 2 studies; I2:0%), uterus didelphys (OR:19.15; 95%CI:15.16-24.18; 3 studies; I2:0%), unicornuate (OR:32.74; 95%CI:6.21-172.67; 3 studies; I2:53%) and bicornuate uterus (OR:17.96; 95%CI:12.19-26.47; 3 studies; I2:27%) (Table SIII; Figure S8).

#### Preterm delivery

The risk of preterm delivery is higher in patients with CUA, both in the global analysis (adjusted OR:4.34; 95%CI:3.59-5.21; 19 studies; I2:56%) and for most anomalies. Estimated OR of preterm birth were 8.91 (95%CI:3.1-25.63) for arcuate uterus (2 studies; I2: 0%), 5.24 (95%CI:1.87- 14.67) for subseptate uterus (2 studies; I2:58%), 4.62 (95%CI:2.43-8.8) for didelphys uterus (7 studies; I2:74%) and 4.45 (95%CI:1.29-15.5) for T-shaped uterus (1 study). Significant Adjusted OR were found for bicornuate (OR:4.9; 95%CI:3.93- 6.11; 7 studies; I2:8%) and unicornuate uterus (OR:3.85; 95%CI:1.84- 8.16; 8 studies; I2:0%). After adjustment, the effect of septate on the risk of preterm delivery became not significant (adjusted OR:1.04 (95%CI: 0.51-2.01; 5 studies; I2:0%) (Table SIII; Figure S9 and Figure S10).

Preterm birth before 34 weeks of gestational age was also more frequent in patients with CUA (OR:5.36; 95%CI:4.29-6.7; 6 studies; I2:12%). The risk of prematurity prior to 34 weeks was increased in presence of didelphys uterus (OR:53.78; 95%CI:5.43-532.94; 1 study) and bicornuate uterus (OR:11.34; 95%CI:1.14-112.75, 1 study) (Crane et al., 2012). No significant effect of septate and unicornuate uterus were detected, and no estimates were available for arcuate, subseptate and T-shaped uterus (Table SIII; Figure S9 and Figure S11).

The rate of preterm delivery before 32 weeks was not affected by CUA (adjusted OR:1.64; 95%CI:0.91-2.97; 6 studies; I2:0%). Septate, didelphys, bicornuate and unicornuate and uterus did not show association with this outcome (Table SIII; Figure S9 and Figure S12). Data to estimate effects of arcuate, subseptate and T-shaped uterus were not available.

#### Caesarean delivery

Compared to women with normal uterus, the caesarean delivery rate was higher in patients with any type of CUA (adjusted OR:7.69; 95%CI:4.17- 14.29; 16 studies; I2:96%) (Table SIII; Figure S13). Caesarean rate was also increased in patients with subseptate uterus (OR:11.27; 95%CI:3.01-42.23; 2 studies; I2:58%), uterus didelphys (adjusted OR:29.9; 95%CI:8.24-126.4; 6 studies; I2:75%), bicornuate (adjusted OR:23.8; 95%CI:10.17-55.7; 6 studies; I2: 46%) and unicornuate uterus (adjusted OR:12.1; 95%CI:5.64-26.5; 6 studies; I2: 0%). Arcuate and septate uterus showed no association with caesarean delivery rate (Table SIII; Figure S14). No estimations for T-shaped effect were available.

#### IUGR/SGA

The risk of IUGR or SGA, considered as a combined outcome, was higher in patients affected by CUA (adjusted OR:50.0; 95%CI:6.11-424; 9 studies; I2:83%) (Table SIII; Figure S15). The rates of IUGR/ SGA were increased in pregnancies of women with subseptate (OR:2.54; 95%CI:1.10-5.89; 2 studies; I2:0%), didelphys (OR:3.82; 95%CI:1.93-7.56; 3 studies; I2:36%), and bicornuate uteri (OR:2.75; CI95%: 1.96-3.86; 4 studies; I2:0%). Arcuate, septate and unicornuate uteri were not associated with this outcome (Table SIII; Figure S16).

#### Foetal and perinatal mortality

Risk of foetal mortality was increased in patients with CUA (OR:2.07; 95%CI:1.56-2.73; 9 studies; I2:10%) (Table SIII; Figure S17). Foetal demise was also more frequent in the presence of uterus didelphys (OR:2.67; 95%CI:1.29-5.51; 3 studies; I2:0%), bicornuate (OR:3.46; 95%CI:2.0-5.99; 3 studies; I2:0%) and unicornuate (OR:2.36; 95%CI:1.23-4.54; 3 studies; I2:0%). Arcuate, subseptate and septate uteri were not associated with increased risk of foetal demise (Table SIII; Figure S18).

Perinatal mortality was higher in women diagnosed with any CUA (OR:3.28; 95%CI:2.01- 5.36; 6 studies; I2:56%) (Table SIII; Figure S19). Specifically, the didelphys (OR:6.69; 95%CI:1.59- 28.15; 2 studies; I2:25%), bicornuate (OR:4.25; 95%CI:1.56-11.6; 2 studies; I2:0%) and unicornuate uterus (OR:3.05; 95%CI:1.75-5.31; 3 studies; I2:0%). No association was found between arcuate, septate, subseptate uterus and increased perinatal mortality (Table SIII; Figure S20).

Analyses of funnel-plot and results of Begg’s and Egger’s tests, performed, when necessary, did not reveal relevant risk of reporting bias.

## Discussion

### Summary of main results

This meta-analytic review supports the association between CUA and adverse obstetrical and perinatal outcomes. Considering the different types of CUA individually, most frequent defects can be classified as U1 (T-shaped), U2 (septate or subseptate), U3 (bicorporate) and U4 (categories of ESGE classification were associated to relevant adverse outcomes. Both septate and subseptate uterus, as well as bicornuate and didelphys uterus, increased risks of miscarriage, preterm birth, foetal malpresentation at delivery, IUGR and need for caesarean delivery. By contrast, arcuate and unicornuate uterus were associated with a significantly lower number of adverse outcomes.

### Comparison with previous studies

Our meta-analysis included 32 studies, which is more than those selected by Chan et al. (2011a) and Venetis et al. (2014), although less than those included in the 2021 Kim’s meta-analysis. Twelve of the studies selected by Kim et al. (2021) were not included in our meta-analysis: one was published out of time limits (Forde et al., 1978) while the other eleven did not achieve the minimum score on the AHRQ-adapted NOS scale (Acién, 1993; Fox et al., 2014; Liang and Hu, 2010; Maneschi et al., 1995; Neal et al., 2019; Ravasia et al., 1999; Shuiqing et al., 2002; Sorensen and Trauelsen, 1987; Woelfer et al., 2001; Zhang et al., 2010; Zupi et al., 1996). Our meta-analysis includes seven studies not considered by Kim et al. (2021) (Cai et al., 2021; Crane et al., 2012; Chen et al., 2018; Lu et al., 2021; Marianna et al., 2022; Qiu et al., 2022; Surrey et al., 2018).

We have detected a significantly increased risk in all the outcomes analysed, with the exception of ectopic pregnancy. We have identified a higher rate of caesarean delivery and intrauterine foetal death, which is consistent with Kim et al. (2021). Our results also support an increased risk of IUGR/ SGA, in line with Kim et al. (2021) but in contrast to Venetis et al. (2014). In our analysis, the pooled effects for several outcomes affected by high levels of statistical heterogeneity, which were not reduced by meta-regression, and that are similar to those estimated in Kim’s in his meta-analysis (Kim et al., 2021).

Canalisation anomalies has been consistently associated with increased risk of miscarriage, as concluded in our meta-analysis and those by Chan et al. (2011a), Venetis et al. (2014), and Kim et al. (2021). According to our results, septate uterus was associated with second trimester but not with first trimester miscarriage, in contrast with the estimation of previous meta-analysis. Our estimation on association of septate uterus on first trimester miscarriage risk is based in pooled results of three good quality studies, which totalled 612 events and 1335 patients. On the contrary, previous meta-analysis included studies performed on small samples and/or excluded from our criteria by low quality scoring. For subseptate uterus, we identified only an increased risk of second trimester miscarriage, contrary to Kim et al. (2021).

Bicornuate uterus is the unification defect most commonly associated with risk of miscarriage, as our study and those of Chan et al. (2011a), Kim et al. (2021), and Venetis et al. (2014) reported. With regard to the pathogenic implication of each type of defect in gestational loss, it should be recalled that bicornuate uterus and the septate/ subseptate uterus share to some extent certain similar characteristics that may explain their causal association with pregnancy loss, such as reduced volume and distensibility of the uterine cavity or abnormal vascularization (Venetis et al., 2014). None of the meta-analyses have found a correlation between uterus didelphys and the risk of miscarriage. Unicornuate uterus seems related to miscarriage according to Venetis et al. (2014), and with first trimester miscarriage as described Chan et al. (2011a).

We found no association between arcuate uterus and risks of miscarriage, which differs from what was previously reported by Chan et al. (2011a), Kim et al. (2021), Venetis et al. (2014). However, this result could be biased by the difficulty in discriminating arcuate uterus from normal or subseptate uterus, due to changes in diagnostic criteria and to differences in clinical imaging accuracy. Therefore, it must be assumed that a proportion of uterus classified as arcuate in the included studies would be considered normal according to current diagnostic criteria. Finally, in our analysis T-shaped uterus markedly increased the risk of first or second trimester miscarriage.

Ectopic pregnancy was more frequent in patients with septate uterus (derived from a single study), which is different from Kim et al. (2021) review, which includes 11 studies on this item (10 of them excluded from our meta-analysis).

Kim et al. (2021) identified an increased risk of placental abruption for all types of CUA, and Venetis et al. (2014) only for arcuate and septate uterus. We found an increased risk of placental abruption only for subseptate and bicornuate uterus. However, as it was obtained from a single study (Takami et al., 2014), it should be considered with caution.

Risk for combined outcome PROM/PPROM from our data revealed a significant association with bicornuate uterus. This result is comparable with that obtained by Venetis et al. (2014), who found increased risk of PROM for arcuate and septate uterus, and by Kim et al. (2021), in whose analysis PPROM rate was increased for all types of CUA.

We found that the preterm delivery rate is increased in most CUA. Previous meta-analyses identified an increased risk of prematurity for subseptate, unicornuate, bicornuate and didelphys uterus (Chan et al., 2011a; Kim et al., 2021; Venetis et al., 2014). Arcuate uterus was not associated with preterm delivery risk according to Chan, Venetis and Kim studies (Kim et al., 2021; Venetis et al., 2014; Chan et al.,2011a), but showed a strong association in our study (OR:8.91; 95%CI:3.10- 25.63). The estimate of Kim et al. (2021) is based on ten studies, eight of which were not included in our synthesis. Septate uterus increases the risk of preterm delivery according to the three previous meta-analyses. This risk was not significant when adjusted by meta-regression, as it depended on the design of the selected studies. Risk of preterm delivery <34 weeks was significantly associated with unification defects (bicornuate and didelphys uterus). Only Venetis et al. (2014) has estimated this correlation, and found an association with most CUA, but not with arcuate uterus. Although never analysed in the past, we did not find correlation between CUA and prematurity <32 weeks with septate uterus or unification defects.

Regarding IUGR/SGA, the three previous meta- analyses (Chan et al., 2011a; Kim et al., 2021; Venetis et al., 2014) found an association with unification defects, especially with didelphys and bicornuate uteri, which is consistent with our results. Canalization defects are less consistently associated with this outcome. Subseptate uterus increases the risk of IUGR/SGA both according to Kim’s study and ours, although Venetis estimated an increased risk of IUGR only for septate uterus (Venetis et al., 2014).

Concerning delivery and postnatal events, malpresentation at delivery is the most frequently reported adverse outcomes for all types of CUA. Caesarean delivery is more frequent in patients carrying canalization defects, as concluded meta- analysis from Kim et al. (2021) and our study. Unification defects also increase the risk of caesarean section, as also by Kim et al. (2021). Chan et al. (2011a) and Venetis et al. (2014) did not consider this outcome. Finally, our results support an increase of risk of foetal mortality in patients with CUA due to unification disorders, while Kim et al. (2021) identified this increased risk only in patients with unicornuate uterus. Perinatal mortality is also associated with unification defects. In our study all the unification defects had increased perinatal mortality, whereas Kim’s meta-analysis detected this increase for unicornuate, bicornuate and septate uterus (Kim et al., 2021).

### Strengths and limitations

Strengths of our study derive from the strict selection criteria. This meta-analysis is the first to estimate pooled effects of T-shaped uterus on pregnancy outcomes obtaining estimates on relevant outcomes (miscarriage, ectopic pregnancy, and prematurity). Our study also provides the first estimation of CUA effects on preterm delivery <32 weeks. Additionally, meta-regression models were applied as an effort to analyse and control the observed heterogeneity.

Retrospective design of most included studies and differences in population of interest, sample sizes, procedures applied for the diagnosis –such as hysterosalpingography or 2D ultrasound–, characteristics of non-exposed patients and design of included studies should be considered as limitations. In addition, classification categories used in includes studies do not correspond with more recent and widely accepted classification schemes of CUA. We have not performed the re-classification of the exposure categories into actual classifications, to avoid the risk of bias potentially associated. Certain outcomes, such as placental abruption and preterm delivery, have been analysed from single studies. Furthermore, several of the ‘effect’ estimates present high levels of statistical heterogeneity, which have not been substantially reduced by meta-regression. Specifically, 30 of the 71 estimates of global effects were affected by high statistical heterogeneity (I2>50%), despite the 10 adjustments.

### Implications for clinical practice

The accurate estimation of risks associated with a specific CUA requires a precise diagnosis and an appropriate standardised classification. In clinical practice, 3-D ultrasound constitutes actually the first- choice image assessment of CUA. In certain cases, complementary tests such as MRI or hysteroscopy may be necessary. There is increasing evidence about the usefulness of hysteroscopic metroplasty in reducing the risk of miscarriage by correction of septate and subseptate uterus (Carrera et al., 2022; Jiang et al., 2023), as well as dysmorphic uterus (Garzon et al., 2020). The surgical treatment of unification defects is more complex with no evidence of improving perinatal prognosis.

### Implications for research

Most studies diagnose and classify CUA according to the first version of the AFS classification. It may be of interest to re-analyse the reproductive risks associated with CUA using the ESHRE/ ESGE or ASRM revised classification, which may help to better estimate the risks associated with these characterised anomalies. The development of prospective studies that apply the most recent classifications are needed.

## Conclusions

CUA are associated with an increased risk of complications in early and late pregnancy. Complications associated with most of the CUA were preterm delivery, malpresentation at delivery, and caesarean delivery. Moreover, our results do not clearly define a profile of preferential association between type of Müllerian defects and category of complications.

## Supplementary material

Table SI Included studies.

Table SII Not included studies and reasons for exclusion.

Table SIII Summary of estimated effects of CUA on pregnancy and neonatal outcomes.

Figure S1 Forest plot of individual and pooled effects on ectopic pregnancy of all CUA

Figure S2 Forest plots of individual and pooled effects on ectopic pregnancy by type of CUA.

Figure S3 Forest plot of individual and pooled effects on placental abruption of all CUA (combined).

Figure S4 Forest plots of individual and pooled effects on placental abruption by type of CUA.

Figure S5 Forest plot of individual and pooled effects on PROM/PPROM of all CUA (combined).

Figure S6 Forest plots of individual and pooled effects on PROM/PPROM by type of CUA.

Figure S7 Forest plot of individual and pooled effects on fetal malpresentation at delivery of all CUA (combined).

Figure S8 Forest plots of individual and pooled effects on fetal malpresentation at delivery by type of CUA.

Figure S9 Forest plots of individual and pooled effects on preterm delivery (A) preterm delivery < 34 wweks (B) and preterm delivery < 32 weeks of all CUA (combined).

Figure S10 Forest plots of individual and pooled effects on preterm delivery by type of CUA.

Figure S11 Forest plots of individual and pooled effects on preterm delivery < 34 weeks by type of CUA.

Figure S12 Forest plots of individual and pooled effects on preterm delivery < 32 weeks by type of CUA.

Figure S13 Forest plot of individual and pooled effects on cesarean delivery of all CUA (combined).

Figure S14 Forest plots of individual and pooled effects on cesarean delivery by type of CUA.

Figure S15 Forest plot of individual and pooled effects on IUGR/SGA of all CUA (combined).

Figure S16 Forest plots of individual and pooled effects on IUGR/SGA by type of CUA.

Figure S17 Forest plot of individual and pooled effects on fetal mortality of all CUA (combined).

Figure S18 Forest plots of individual and pooled effects on fetal mortality by type of CUA.

Figure S19 Forest plot of individual and pooled effects on perinatal mortality of all CUA (combined).

Figure S20 Forest plots of individual and pooled effects on perinatal mortality by type of CUA.
