# Supplementary material for: Impact of congenital uterine anomalies on obstetric and perinatal outcomes: systematic review and meta-analysis
Source: Facts Views Vis Obgyn. 2024 Mar 28;16(1):9–22. doi: 10.52054/FVVO.16.1.004 (PMC11198883; doi:10.52054/FVVO.16.1.004)
Supplement: Table SI — Included studies. [file FVVinObGyn-16-9-ts001.pdf]

Table SI. — Included studies.

Study	*Design and study period*	*Setting*	*Population of interest*	*Exclusion criteria*	*Müllerian abnormalities considered*	*Exposed patients (number and characteristics)*	*Non-exposed patients (number and characteristics)*	*Classification system applied*	*Method of diagnosis*	*Outcomes considered*	*Control for confounders*	*Sub-analyses*
Ben-Rafael 1991	Retrospective cohort study (study period not reported)	Tertiary hospital	Women who underwent HSG for history of primary or secondary infertility, or RPL	Arcuate and subseptate uterus	Unicornuate, bicornuate and dideplhys uterus	67 patients with mullerian anomalies: 58 bicornuate, 5 unicoruate and 4 didelphys uterus	130 patients with normal shaped uterus in HSG, quasi-randomly selected among patients with same indcations for HSG	AFS	HSG	Total pregnancies, spontaneous miscarriage, induced abortion, immature delivery (20-26 weeks), premature delivery, term birth, intrauterine fetal demise, bleeding during pregnancy, PPROM, postpartum haemorrhage, placental retention, type of presentation	Maternal age, parity	Indication for HSG (infertility or RPL)
Cooney 1998	Retrospective cohort study (study period not reported)	Tertiary hospital	Women with singleton ongoing pregnancies at 1st trimester US	Equivocal US images and a lack of independent confirmation	Septate or subseptate uterus	22 women in whom a uterine duplication abnormality (septate or subseptate uterus)	66 with patients with normal appearing uteri	AFS	US, HSG, hysteroscopy or laparotomy	Pregnancy loss, liveborn infants, delivery < 34 and ≥ 34 weeks, cesarean section, 1 minute Apgar <7, 5 minute Apgar <7	Maternal age, indication for US scan	None
Leible 1998	Prospective cohort study (1993-96)	Tertiary university hospital	Women with clinical pregnancies who delivered during study period	Not reported	Septate, bicornuate and didelphys uterus	CUA: 7 septate uterus, 7 bicornuate and 1 didelphys.	Pregnant women with a normal uterine cavity randomly selected from those attending for US (30)	AFS	Hysterography, US and surgical findings	Birth weight, gestational age at delivery, IUGR, preeclampsia, preterm birth, stillbirth	Gestational age	None

Erez 2007	Retrospective cohort study (1988-2002)	Tertiary university hospital	All patients with a previous cesarean section that attempted vaginal birth after cesarean section during the study period.	Patients with multiple pregnancies, more than 1 previous cesarean section or known congenital and/ or chromosomal fetal anomalies	Septate, arcuate, unicornuate, didelphys and bicornuate uterus	165 patients with müllerian anomalies (28 septated, 32 arcuate, 15 unicornis, 13 didelphys and 77 bicornuate uterus)	5406 patients with normal uterus	AFS	Diagnostic workout and surgical findings	Hydramnios, oligohyrdramnios, PROM, PPROM, preterm delivery, arrest of labor, placental abruption, severe preeclampsia, cord prolapse, caesarean section, 1 and 5 minute Apgar, birth-weight, uterine rupture	Malpresentation, cord prolapse, arrest of labor, NRFHR, placental abruption, hydramnios, maternal age, preterm delivery, PPROM, maternal gestational diabetes, preeclampsia, LGA, SGA	Indication for primary CS according to specific type of Müllerian duct anomalies. Comparison of pregnancy outcome of the study groups according to fetal presentations
Zlopasa 2007	Retrospective cohort study (1997-2000)	Tertiary perinatal centre	General population of pregnant women	Twin gestations, chorioamnionitis, presence of submucosal myomas, fetal chromosomopathy, maternal diabetes, IVF pregnancies	Arcuate, bicornuate (partial and total), didelphys, unicornuate, subseptate and septate uterus	130 women with uterine anomalies (246 pregnancies): 13 arcuate, 78 partial bicornuate, 13 total bicornuate total, 43 didelphys, 8 unicornuate, 31subseptate and 60 septate uterus.	182 randomly selected women with a previously confirmed normally shaped uterus (379 pregnancies)	Modified AFS classification	Previous surgery findings, sonohysterography, laparoscopy with hysterography, or hysteroscopy	Bleeding during pregnancy, pregnancy, delivery, fetal asphyxia, placental abruption, Apgar score 1 min, Apgar score 5 min, miscarriage rate, term delivery, preterm delivery, birth weight, gestational age at delivery, fetal malposition, IUGR, cesarean section, mortality (fetal, early neonatal, perinatal).	Age, parity	Type of uterine abnormality
Ban-Frangez 2009	Retrospective cohort study (1993-2004)	Tertiary university hospital	Women who had conceived following an IVF/ICSI procedure during the period	Not reported explicitly	Subseptate and septate uterus	Pregnant women after IVF or ICSI prior to hysteroscopic resection of a large (n = 12) or small partial uterine septum (n = 19)	Women without uterine anomalies with a singleton intrauterine pregnancy and visible foetal heartbeat (n = 62)	AFS	2D vaginal US without intrauterine saline infusion, hysteroscopy	Miscarriage	Age, BMI, stimulation protocol, use of IVF or ICSI and infertility causes	None

Sugiura-Ogasawara 2010	Retrospective cohort study (referred by authors as case-control study) (1986-2007)	Tertiary university-affiliated hospital	Patients with a history of ≥ 2 consecutive miscarriages who became subsequently pregnant after a systematic assessment of RPL	Structural chromosomal abnormalities detected during study of causes of RPL	Septate and bicornuate uterus	5 patients with septate uterus and 37 patients with bicornuate uterus (53 pregnancies)	1528 women with normal uterus (3433 pregnancies)	Modified AFS classification and Tompkin's Index	Laparoscopy, laparotomy and/or MRI	Live birth rate per pregnancy Cumulative live birth rate	Maternal age, number of previous miscarriages, number of previous live births, number of previous live stillbirths	Type of uterine abnormality (septate and bicornuate)
Saravelos 2010	Retrospective cohort study (study period not reported)	Tertiary hospital	Women with recurrent RPL (three or more consecutive pregnancy losses prior to 24 weeks of gestation)	Pregnancies in which patients had received medical treatment (e.g. low molecular weight heparin, acetyl-salicylic acid, steroids) or surgery (e.g. septotomy, Strassman's metroplasty, cervical cerclage)		56 patients with a specific CUA (diagnosed by hysteroscopy/ laparoscopy) and no other identifiable cause of RM formed the study group	107 Women with normal investigations for identifiable causes of RPL (unexplained RM) formed the control group (n = 107)	Modified AFS classification	2D US , HSG and hysteroscopy/laparoscopy	Biochemical miscarriage, 1st and 2nd trimester miscarriage, ectopic pregnancy, live birth	Maternal age, height, weight, BMI and gravidity	Type of uterine abnormality
Tomazevic 2010	Retrospective cohort study (1993-2004)	Tertiary university hospital	Infertile patients treated with IVF/ICSI ET	Not reported	Septate/subseptate and arcuate uterus	113 ET in patients with septate-subseptate uterus and 176 ET in patients with arcuate uterus	578 ET in patients with normal uterus	Modified AFS classification	2D US	Pregnancy, live birth	Length of uterine septum, maternal age, classic IVF or ICSI, number and quality of embryos transferred.	Length of uterine septum (septate-subseptate or arcuate),
Hua 2011	Retrospective cohort study (1990-2008)	Tertiary care medical centre	Patients with singleton pregnancies undergoing routine anatomic survey	Multiple pregnancy, lack of information in database about exposure, outcomes or covariates	Bicornuate, didelphys, septum, unicornuate and other abnormal uteri.	203 patients with presence of a uterine anomaly diagnosed prior to pregnancy or at initial ultrasound evaluation of uterine anatomy	66753 patients with normal uterine morphology	Modified AFS classification	US	Spontaneous preterm birth (<34 and <37 weeks), PPROM, breech presentation, cesarean delivery IUGR	History of preeclampsia. maternal renal disease, chronic hypertension, gestational diabetes, stillbirth, preterm birth, race	None
Jayaprakasan 2011	Prospective cohort study (2005-2009)	Tertiary centre Fertility Unit	Infertile patients referred for treatment	Difficult delineation of the shapes of the uterus, or uterine cavity distortion by fibroids	Arcuatus,, septate, unicornuate, subseptate, bicornuate and T-shaped uterus	184 patients with abnormal uterus: 164 arcuatus, 7 septate, 6 unicornuate, 5 subseptate, 1 bicornuate, 1 T-shaped.	1201 patients with normal cavity uterus	AFS classification	2D and 3D US	First Trimester miscarriage and ongoing pregnancy (not defined)	Maternal age, basal FSH, AFC	Type of müllerian anomaly

Crane 2012	Retrospective cohort study (2000-08)	Three tertiary care centres	Pregnant women with singleton pregnancies who delivered	Multiple pregnancy, previous cervical cerclage or septum resection	Bicornuate, unicornuate, didelphys, and septate uterus,	52 uterine anomalies: 35 bicornuate uterus, 13 uterus didelphys, 2 septum, 2 unicornuate uterus)	Women without a uterine anomalies, and without a history of preterm delivery or treatment for cervical dysplasia, (122)	AFS	HSG, US, hysterosonography, CT, MRI, hysteroscopy/ laparoscopy or laparotomy	Preterm birth, gestational age at delivery, birth weight, Apgar score, neonatal intensive care unit admission, cord arterial pH, perinatal morbidity and mortality, type of delivery and need for induction.	Gestational age, cervical length	Type of müllerian anomaly
Takami 2014	Retrospective cohort study (2000-2012)	Tertiary care university centre	Women who delivered a live singleton baby after 22 gestational weeks	Pregnancies affected congenital abnormalities and prior uterine surgery	Arcuate, bicornuate, subseptate, septate and didelphic uterus	Patients with congenital uterine anomalies and without prior uterine surgery (n=80) including arcuate (n=4), unicornuate (n=3) bicornuate (n=25), subseptate (n=27), septate (n=6) and didelphic uterus (n=15)	5763 women with normal uterine morphology, who received prenatal care starting early in pregnancy and delivered a live singleton baby after 22 gestational weeks during the study period	AFS	2D US, MRI, hysteroscopy and surgical findings	Preterm birth, fetal malpresentation, caesarean delivery, placental abruption, SGA, LGA	Age, parity, gravidity	Number of cervical orifices (one vs two)

Hiersch 2016	Retrospective cohort study (2007-2014)	Tertiary university hospital	Women who delivered during study period	Women who underwent any surgical treatment of uterine anomalies, who delivered before 24 weeks of gestation and pregnancies with uncertain pregnancy dating or those complicated by stillbirth or major fetal anomalies	Bicornuate, septate, unicornuate and didelphyc uterus.	243 women with uterine congenital anomalies: 156 bicornuate uterus, 38 septate uterus, 27 unicornuate uterus and 22 didelphic uterus.	Women with normal uterus who delivered during study period matched by age (±2 years), number of fetuses and parity (total number of prior deliveries ≥24 weeks of gestation) in a 1:2 ratio (486)	AFS	HSG, US, hysterosonography, computed tomography, magnetic resonance imaging, hysteroscopy, laparoscopy or laparotomy	Oligohydramnios, PROM, PPROM, Preterm birth <37 weeks, <34 weeks and <32 weeks, Induction of labor, Elective caesarean section, operative vaginal delivery, Cesarean delivery, post-partum haemorrhage, retained placenta, post-partum fever, birthweight > 4000 g, mmall for GA, ppgar 5 min < 7, umbilical artery cord pH < 7.10, neonatal sepsis, neonatal death	Age, number of fetuses and parity	None
Li 2017	Retrospective cohort study (2012-2014)	Tertiary centre Fertility Unit	Infertile outpatients who successfully achieved pregnancy	Age > 40 years, BMI <18 or >28 kg/m2, missing one of the ovaries, donor oocytes, PGD/PGS, parental chromosomal abnormalities, embryo reduction (spontaneous or elective), triplet pregnancies, induced labour for fetal anomalies, uterine fibroids or polyps distorting the endometrial cavity	Unicornuate uterus	238 pregnant patients with unicornuate uterus from 455 who received IVF treatment	818 pregnant patients with normal uterus from 1484 who received IVF treatment	ESHRE/ESGE classification	US, HSG, hysteroscopy and/or laparoscopy	Clinical pregnancies, early pregnancy loss, late miscarriage, ectopic pregnancy, preterm delivery, very preterm birth, term delivery, live birth, stillbirth, perinatal mortality, LBW, VLBW	Maternal age, BMI, previous miscarriage, in- fertility type, insemination methods, transfer cycle, number of retrieved oocytes and endometrial thickness on transfer day, infertility duration, cause of infertility, FSH, number of transferred embryos and 14-day HCG.	Number of fetuses (all, single and twin pregnancies)

Ozgur, 2017	Retrospective cohort study (2009-2015)	Reproductive medicine centre	Infertile patients undergoing first IVF treatment with fresh or frozen embryo transfer.	Second or later attempts of IVF or FET	Unicornuate uterus	50 cycles as treatment of women with unicornuate uterus	100 matched cycles in women with normal uterus, randomly selected by embryo transfer strategy, woman's age, number of oocytes retrieved and antral follicular count	AFS	2D transvaginal US, HSG, saline-infused sonography or hysteroscopy or laparoscopy	Biochemical pregnancy, clinical pregnancy, ongoing pregnancy, pregnancy loss, implantation rate	Embryo transfer strategy age (fresh or frozen embryos), number of oocytes retrieved and antral follicular count	None
Mastrolia 2017	Retrospective cohort study (1988-2013)	Tertiary university hospital	Women carrying a singleton pregnancy who delivered during study period	Not reported	Bicornuate uterus	444 pregnancies in women with bicornuate uterus	279,662 pregnant women with normal uterus	Modified AFS classification	Not reported	Mild or severe preeclampsia, poly/ oligohydramnios, PROM, cervical insufficiency, vaginal bleeding, preterm contractions, macrosomia, placental abruption, placenta previa, non-progressive labor, cord prolapse, knots or laces, preterm delivery, postpartum haemorrhage, IUGR, abnormal presentation, mode of delivery, perinatal mortality. Apgar 1 min <7 and <5, Apgar 5 min <7 and <5, birth weight, gestational age at delivery, cord pH, base excess.	Maternal age, parity, grand multiparity, ethnicity, recurrent abortions (only for cervical insufficiency analysis)	None

Cahen-Peretz 2017	Population-based retrospective cohort study (1991-2013)	Tertiary university hospital	Women who delivered during observation period	Multifetal pregnancies, unknown gestational age, gestational age of less than 24 weeks upon delivery, and fetal congenital malformations	Septate uterus, bicornuate uterus, unicornuate uterus, uterus didelphys, and arcuate uterus	Septate uterus, bicornuate uterus, unicornuate uterus, uterus didelphys, and arcuate uterus (n=1251)	Patients who were not diagnosed with Müllerian anomalies	Modified AFS classification	US, HSG, hysterosonography, MRI, hysteroscopy, laparoscopy, or laparotomy	Poly/oligo-hydramnios, placenta previa, retained placenta, placental abruption, vasa previa, macrosomia, IUGR, chronic hypertension, preeclampsia, eclampsia, gestational diabetes, anemia, PROM, meconium stained amniotic fluid, postpartum haemorrhage, shoulder dystocia, uterine rupture, pathological presentation, breech presentation, vaginal birth, assisted birth, caesarean section, peripartum hysterectomy, low Apgar 1 min (<7), low Apgar 5 min (<7), birthweight (g), SGA, LBW, VLBW, perinatal mortality.	Maternal age, parity, gestational age at delivery, RPL, previous cesarean section, preeclampsia, gestational diabetes, fertility treatment, SGA, hypertensive disorders, gestational diabetes, previous cesarean section	Type of Müllerian anomalies: uterus didelphys/ other müllerian anomalies/ no anomalies

Mastrolia, 2018	Retro-spective popula-tion-based cohortstudy (1988-2013)	Tertiary university hospital	General population who delivered during study period	Patients with multiple pregnancies or missing data were excluded from the study	Congenital uterine malformations	Women with congenital uterine malformations (n = 1099)	Women with anatomically normal uterus s (n = 279662).	AFS	Not specifically reported (workup for infertility or recurrent pregnancy loss, accidental finding during pregnancy, or noticed at the time of cesarean delivery).	Mild or severe preeclampsia, poly/oligohydramnios, PROM, cervical insufficiency, vaginal bleeding, preterm contractions, macrosomia, placental abruption, placenta previa, non-progressive labor, cord prolapse, knots or laces, preterm delivery, postpartum haemorrhage, IUGR, abnormal presentation, mode of delivery, perinatal mortality. Apgar 1 min <7 and <5, Apgar 5 min <7 and <5, birth weight, gestational age at delivery, cord pH, base excess.	Maternal age, parity, grand multiparity, ethnicity, recurrent abortions (only for cervical insufficiency analysis)	None
Ples, 2018	Retrospective cohort study (2016-17)	Tertiary university hospital	Infertile patients undergoing diagnostic workup and IVF treatment	Associated uterine pathology (one or more polyps, synechiae, or submucosa myomas) and ultrasound image not sufficient for a definitive diagnosis	Dysmorphic uterus (U1c), incompletely septate uterus (U2a), and completely septate uterus (U2b)	52 patients diagnosed with uterine congenital anomalies receiving IVF treatment: dysmorphic uterus (class U1c; 18 patients), incompletely septate uterus (class U2a; 17 patients), and completely septate uterus (classU2b; 10 patients)	148 patients with normal uterus receiving IVF treatment	ESHRE/ESGE	2D and 3D US	Miscarriage, clinical pregnancy, ongoing pregnancy	Baseline characteristics of patients regarding infertility and IVF treatment	Type of Müllerian anomalies
Prior 2018	Prospective cohort study (2009-2015)	Reproductive medicine centre	Infertile patients recruited since initial assessment for subfertility and undergoing IVF treatment with fresh ET)	Impossibility of a definitive diagnosis caused by presence of fibroids, intrauterine device or polyps distorting the cavity, history of Asherman's syndrome or previous hysteroscopic surgery or poor quality of images	Arcuate, subseptate, septate, bicornuate, unicornuate and didelphys uterus	432 patients with congenital uterine abnormalities: 387 arcuate, 16 subseptate, 11 septate, 4 bicornuate, 13 unicornuate, 1 didelphys.	1943 patients with normal uterus	Modified AFS classification	3D US	Live birth rate, multiple live birth. clinical pregnancy, preterm birth (<37, < 34, and <32 weeks)	Covariates with significant differences between exposed and non-exposed patients: parity, BMI and number of embryos transferred	Type of abnormality regarding live birth, clinical pregnancy

Surrey 2018	Retrospective cohort study (2014)	Tertiary care assisted reproduction centre	Patients undergoing in vitro fertilization and euploid ET after chromosome screening	Use of donor oocytes or gestational carrier, evidence of other endometrial cavitary abnormalities, fundal indentation <4 mm.	Arcuate uterus	Arcuate uterus (83 FET cycles performed in 76 patients)	378 frozen-ET cycles performed in 354 patients without uterine abnormalities	Modified AFS classification	2D and 3D transvaginal US and hysteroscopy	Implantation rate, live birth rate, biochemical pregnancy rate, miscarriage rate	Maternal age, AMH, basal FSH, AFC, biopsied blastocysts, euploid blastocysts, transferred blastocysts	None
Chen 2018	Retrospective cohort study (2012-16)	University and university-affiliated reproductive medicine centres	Women who underwent first IVF/ICSI cycle in study centres.	Uterine malformations different than unicornuate uterus class IVb , endometrial lesions (polyps, endometrial hyperplasia, intrauterine adhesions), sonographic features of adenomyosis, parental chromosomal abnormality, donor oocytes, PGD/PGS, cancelled IVF cycle prior to ET	Unicornuate uterus class IVb (isolated hemi-uterus without functional rudimentary cavity)	Patients with unicornuate uterus (n=342)	1026 controls randomly selected, matched in a ratio of 1:3 by age, BMI, cause of infertility, and number of embryos transferred	ESHRE/ESGE classification	3D transvaginal US, hysteroscopy with or without laparoscopy or MRI	Cumulative live birth rate, implantation rate, miscarriage rate, cumulative clinical pregnancy rate, clinical pregnancy per transfer cycle and live birth rate per transfer cycle	Maternal age, BMI, basal FSH, infertility duration, parity and main cause of infertility, GnRH analogue protocol, rate of ICSI procedure, total dose of gonadotropins, E2, LH and Progesterone on HCG day, number of collected oocytes and number of available embryos.	Reproductive outcomes calculated for fresh ET cycle: (cleavage day-3 or blastocyst) and for ET cycles (cleavage day-3 ET cycles or blastocyst). Cumulative reproductive outcomes from one complete ART cycle including fresh and frozen-thaw ETs
Chen 2019	Retrospective cohort study (2009-11)	University and university-affiliated reproductive medicine centres	Infertile patients treated with IVF or ICSI	Oocyte donor treatment cycles, abnormal uterine bleeding, endometrial fibroids or polyps, intrauterine adhesion, premature ovary insufficiency, polycystic ovary syndrome, and history of ≥ 3 consecutive spontaneous miscarriages.	Unicornuate uterus	160 patients with unicornuate uterus who underwent 329 IVF/ICSI cycles	160 randomly selected controls with normal uterus (matched in a ratio of 1:1 by age, BMI, cause of infertility, and number of embryos transferred) who underwent 390 IVF/ICSI cycles	Modified AFS classification	Transvaginal US, HSG and hysteroscopy/laparoscopy	Endometrial thickness on HCG day, oocytes retrieved, biochemical pregnancy, clinical pregnancy, live birth, total pregnancy loss, early miscarriage (12 weeks), term delivery.	Maternal age, BMI, Primary infertility, History of spontaneous abortion (once or twice), Prior ectopic pregnancy, Indication for IVF/ICSI, Antral follicle count, basal FSH, basal LH, number of good quality embryos, fresh ET	Number of foetuses (singleton vs twin)

Ouyang, 2020	Retrospective cohort study (2012-2014)	Reproductive medicine centre	First clinical pregnancies from IVF-ET delivered at ≥ 22 gestational weeks (singleton, twins and embryo selective reduction)	Maternal age ≥ 40 years old; body mass index outside 18–28 kg/m2 range; only one ovary detected; uterine fibroids or polyps distorting the endometrial cavity; received donor oocytes; preimplantation genetic diagnosis/ preimplantation genetic screening; parental chromosomal abnormalities; spontaneous embryo reduction; monochorionic twin or triplet pregnancies; early or late miscarriage; ectopic pregnancy and induced labour	Unicornuate uterus	Pregnant patients with unicornuate uterus (n=206)	Outpatients with normal uterus (314)	AFS	3D transvaginal US	Live birth, preterm delivery, perinatal mortality, cesarean delivery, LBW, VLBW	Infertility type, number of transferred embryos, IVF method, maternal age, basal FSH	Number of fetuses (singleton, twin reduced to singleton and twins)
Cai 2021	Retrospective cohort study (2005-2018)	Tertiary perinatal hospital	Infertile patients who underwent IVF-ET and achieved clinical pregnancy	Asherman's syndrome, uterine fibroid or endometrioma distorting the uterine cavity, untreated hydrosalpinx, previous uterine surgery, cancellation of IVF cycle prior to ET, donor oocytes, PGT, selective/ spontaneous foetal reduction, induced labour for congenital abnormalities.	Uterus didelphys	83 infertile patients with uterus didelphys	249 patients presenting randomly selected and matched by number of gestational sacs (singleton or twin), maternal age (± 1 year), infertility type (primary or secondary), cause of infertility	Modified AFS classification	3D transvaginal US	Miscarriage, preterm delivery ectopic pregnancy, live birth, term birth, preterm birth (from 20 to 32 weeks of gestation), perinatal mortality, LBW, VLBW	Maternal age type of infertility (primary or secondary), cause of infertility, fertilization technique (classic IVF or ICSI), endometrial thickness one day before the day ET, number of embryos transferred	Per number of gestational sacs (singleton or twin)

Kong 2021	Retrospective cohort study (2009-2018)	University-affiliated centre of reproductive medicine	Patients treated with first cycle of IVF/ICSI and with subsequent FET cycles	Severe systemic disease, uterine or pelvic disease (severe intrauterine adhesions, uterine adenomyosis, or untreated hydrosalpinx), chromosomal abnormality in the male or female partner, donor oocytes, preimplantation genetic test treatment drop-out or no follow-up information availability	Bicornuate uterus	58 patients with bicornuate uterus	174 women with normal uterus who were randomly selected in accordance with three essential conditions (age, BMI and co-existing PCOS). Additional balancing criteria were applied with a 1:3 exposed/ non-exposed ratio (four of six of following criteria: basal FSH, antral follicular count, cause of infertility, assisted reproduction treatment, controlled ovarian stimulation protocol; and number of embryos transferred).’	Modified AFS classification	Hysteroscopy combined with laparoscopy, surgery or caesarean section (12 patients), or pelvic MRI (1 patient), or 3D US (13 patients), or 2D US combined with hysteroscopy and/or HSG (32 patients).	Cumulative pregnancy rate and Cumulative live birth rate, Implantation rate, biochemical pregnancy, clinical pregnancy, multiple pregnancy (fresh ET cycles only), ectopic pregnancy; 1str and 2nd trimester miscarriage; live birth; preterm delivery; and (10) term delivery, caesarean section; neonatal birth weight; PPROM, placenta praevia, gestational diabetes and hypertensive disorders of pregnancy.	Maternal age, BMI, presence of PCOS (complete balancing) Basal FSH, AFC, cause of infertility, IVF technique, stimulation protocol and number of transferred embryos (relative balancing)	Reproductive outcomes calculated per fresh ET cycle Reproductive outcomes calculated per frozen ET cycle Cumulative reproduction outcomes considering fresh and frozen ET

Lü 2021	Retrospective cohort study (1999-2019)	Tertiary university hospital	Patients with singleton deliveries at ≥28 weeks	Multiple deliveries, singleton deliveries prior to 28 weeks	Unicornuate uterus	44 deliveries in 43 women with unicornuate uterus	367 randomly selected deliveries with normal uterus	Modified AFS classification	US or MRI, endoscopy for various indications, previous abdominal surgery	Preterm delivery rate, breech presentation, cesarean delivery	Multivariate adjustment by covariates which differs between exposed and non-exposed patients: gravidity, parity, nulliparity, IVF pregnancy rate, history of ectopic pregnancy, and incidence of preeclampsia)	Grade of preterm delivery (28 to <32 weeks or 32 to <37 weeks). Etiology of preterm birth (spontaneous or iatrogenic) and Indications of caesarean delivery
Zambrotta, 2021	Retrospective cohort study (2010-20)	Tertiary university hospital	Women with clinical pregnancies who delivered during study period	Women without ultrasonographically confirmed pregnancy, without confirmation or exclusion diagnosis of uterine malformations, who underwent ART cycles or who experienced at least one miscarriage	Didelphys, bicornuate, unicornuate, arcuate, complete septate and uncomplete septate uterus	Pregnant patients with history of one or more pregnancies or current pregnancy with diagnosis of uterine anomalies (n=29)	Women hospitalized for delivery with normal uterine morphology at ultrasound, recruited during the same period (100)	AFS	3D transvaginal US	Preterm birth, fetal malpresentation, and SGA fetus	Logistic regression (age, gestational week, ethnicity and smoking habit)	None

Zhang, 2021	Retrospective cohort study (2008-2019)	Tertiary university hospital	Women who underwent fresh and frozen-thawed cycles of in vitro fertilization/ intracytoplasmic sperm injection-embryo transfer (IVF/ ICSI-ET)	Other types of uterine malformations (mediastinal uterus, bicorne uterus, etc.); diminished ovarian reserve; endometrial lesions; uterine fibroids; adenomyosis; polycystic ovary syndrome; recurrent miscarriage; chromosomal abnormalities.	unicornuate	109 patients with unicornuate uterus Women who underwent fresh and frozen-thawed cycles of in vitro fertilization/ intracytoplasmic sperm injection-embryo transfer (IVF/ ICSI-ET)	2390 patients with normal uterine morphology	Not mentioned	Transvaginal ultrasound, HSG, and hysteroscopy/ laparoscopy	embryo implantation rate, clinical pregnancy rate, miscarriage rate, ectopic pregnancy rate, infant mortality rate, live birth rate, single live birth rate, twins live birth rate, reduction in twin pregnancy rate. Neonatal outcome: delivery method, newborn sex rate, premature birth rate, fetal birth weight, low birth weight infant (LBW) rate Macrosomia rate.	age, type of infertility, fertilization method, number of embryos transplanted, and uterine morphology	none
Marianna 2022	Hospital-based prospective cohort study	Tertiary university hospital	Patients 20–40 years old who underwent 3-dimensional ultrasound of uterine cavity before embryo transfer	Patients with uterine fibroids, uterine cavity deformities due to uterine surgery or synechiae or adenomyosis; with endometrial polyps, history of ovarian surgery, with genetic thrombophilia, hydrosalpinx, or significant obesity (BMI > 35 kg/m2).	U1a (T-shaped), intermediate (T borderline), U1b (infantilis), U2a (subseptate), U2B (septate), U3b (bicorporeal complete) and U5 uterus (aplastic)	Patients with U1a (T-shaped) (n = 27) intermediate (T borderline) uterus (n = 73), Other anomalies (U1b [infantilis], U2a [subseptate], U2b [septate], U3b [bicorporeal complete] and U5 uterus [aplastic]) (n = 22)	Patients with normal uterine cavity (n = 266)	ESHRE/ ESGE	3D transvaginal US	Term deliveries, preterm deliveries, miscarriage, ectopic pregnancy	Age, BMI, endometrial thickness on day of embryo transfer, previous pregnancies, deliveries, miscarriages, past failed IVF attempts, serum AMH and serum FSH.	Type of abnormality

Qiu 2022	Matched controls retrospective cohort study (2008-2019)	Tertiary university hospital	University-affiliated centre of reproductive medicine	Women undergoing their first FET cycles	Bicorporeal, septate and unicornuate uterus	92 bicorporeal uteri, 195 septate uteri and 124 unicornuate uteri.	Patients with normal uterus after routine infertility diagnosis workout, selected within the population-based cohort by mean of propensity score matching (900 controls for bicorporeal uterus group, 1496 controls for septate uterus group and 456 controls for hemi-uterus group	ESHRE/ ESGE and AFS	2D transvaginal US	Biochemical pregnancy, implantation, miscarriage, ectopic pregnancy, clinical pregnancy, live birth, diabetes mellitus, gestational hypertension, preeclampsia and eclampsia, nephritis gravidarum, gestational anaemia, intrahepatic cholestasis, placental abruption, placenta praevia, PROM, preterm birth, LBW, VLBW, ELBW, SGA, neonatal diseases	Age, BMI, duration of infertility, gravidity, parity, presence of different infertility causes, type of endometrial preparation (natural cycles, hormone therapy cycles and stimulated cycles), endometrial thickness on ET day, number of retrieved oocytes, number of frozen cleavage embryos, number of frozen blastocysts, number of embryos transferred and year of treatment	Type of ab-normality

Abbreviations: AFC: antral follicular count; AFS: American Fertility Society; BMI: body mass index; ET: embryo transfer; HSG: HSG; IVF: in vitro fertilization; ICSI; intracytoplasmatic sperm injection; IUGR: intrauterine growth restriction; LGA: large for gestational age fetus; LBW: low birth weight (<2500 g); MRI: magnetic resonance imaging; PCOS: polycystic ovarian syndrome; PGD: preimplantational diagnosis; PGS: preimplantational screening; PGT: preimplantational testing; PPROM: preterm premature rupture of membranes; PROM: premature rupture of membranes; SGA: small for gestational age fetus; US: US; VLBW: very low birth weight (<1500 g); ELBW: extremely low birth weight (<1000 g)
