# Supplementary material for: Impact of congenital uterine anomalies on obstetric and perinatal outcomes: systematic review and meta-analysis
Source: Facts Views Vis Obgyn. 2024 Mar 28;16(1):9–22. doi: 10.52054/FVVO.16.1.004 (PMC11198883; doi:10.52054/FVVO.16.1.004)
Supplement: Table SII — Not included studies and reasons for exclusion. [file FVVinObGyn-16-9-ts002.pdf]

Table SII. — Not included studies and reasons for exclusion.

Study	Design	Reasons for exclusion
Portuondo, 1986	Case-control study	Study design
Sorensen, 1987	Retrospective cohort study	Lack of control for potential confounders (not granted in comparability domain of NOS score and was considered not eligible following AHRQ standards)
Rogers, 1985	Descriptive study	Non-comparative study
Ben-Rafael, 1990	Retrospective cohort study	Double publication (same data analysed in Ben-Rafael, 1991)
Acien, 1993	Retrospective cohort study	Not normal controls (non-exposed patients have normal uterus but other congenital genitourinary abnormalities).
Fedele, 1995	Descriptive study	Non-comparative study
Maneschi, 1995	Retrospective cohort study	Lack of control for potential confounders (not granted in comparability domain of NOS score and was considered not eligible following AHRQ standards)
Lavergne, 1996	Retrospective cohort study	Lack of control for potential confounders (not granted in comparability domain of NOS score and was considered not eligible following AHRQ standards)
Colacurci, 1996	Retrospective cohort study	Studied population out of scope
Zupi, 1996	Descriptive study	Non-comparative study
Raga, 1997	Descriptive study	Non-comparative study
Ravasia, 1999	Retrospective cohort study	Lack of control for potential confounders
Grimbizis, 2001	Systematic review	Non-primary study
Woelfer, 2001	Prospective cohort study	Lack of control for potential confounders (not granted in comparability domain of NOS score and was considered not eligible following AHRQ standards)
Shuiqing, 2002	Prospective cohort study	Not normal controls (non-exposed patients have normal uterus but other congenital genitourinary abnormalities).
Salim, 2003	Case-control study	Study design
Akar, 2005	Descriptive study	Non-comparative study
Airoldi, 2005	Retrospective cohort study	Comparison out of scope
Tomazevic, 2007	Retrospective cohort study	Studied population out of scope
Sendag, 2010	Retrospective cohort study	Studied population out of scope
Liang, 2010	Retrospective cohort study	Lack of control for potential confounders (not granted in comparability domain of NOS score and was considered not eligible following AHRQ standards)
Zhang, 2010	Retrospective cohort study	Lack of control for potential confounders (not granted in comparability domain of NOS score and was considered not eligible following AHRQ standards)
Tonguc, 2011	Retrospective cohort study	Studied population out of scope
Ghi, 2012	Descriptive study	Non-comparative study
Chen, 2013	Retrospective cohort study	Studied population out of scope
Jaslow, 2013	Case-control study	Not included design
Acien, 2014	Retrospective cohort study	Studied population out of scope

Fox, 2014	Retrospective cohort study	Lack of control for potential confounders (cohorts differs in several baseline variables with could act as confounders; no adjustment was performed). The study was not granted in comparability domain of NOS score and was considered not eligible following AHRQ standards.
Tofoski, 2014	Retrospective cohort study	Studied population out of scope
Sugiura-Ogasawara, 2015	Retrospective cohort study	Studied population out of scope
Elsokkary, 2018	Retrospective cohort study	Studied population out of scope
Gabbai, 2018	Case-control study	Not included design
Ludwin, 2018	Letter to editor	Non suitable for analysis
Alonso-Pacheco, 2019	Retrospective cohort study	Studied population out of scope
Fox, 2019	Retrospective cohort study	Studied population out of scope
Neal, 2019	Retrospective cohort study	Lack of control for potential confounders (cohorts differs in several baseline variables with could act as confounders; no adjustment was performed). The study was not granted in comparability domain of NOS score and was considered not eligible following AHRQ standards.
Ridout, 2019	Retrospective cohort study	Comparison out of scope
Hynes, 2021	Descriptive study	Non-comparative study
