# Supplementary material for: Impact of congenital uterine anomalies on obstetric and perinatal outcomes: systematic review and meta-analysis
Source: Facts Views Vis Obgyn. 2024 Mar 28;16(1):9–22. doi: 10.52054/FVVO.16.1.004 (PMC11198883; doi:10.52054/FVVO.16.1.004)
Supplement: Table SIII — Summary of estimated effects of CUA on pregnancy and neonatal outcomes. [file FVVinObGyn-16-9-ts003.pdf]

Table SIII. — Summary of estimated effects of CUA on pregnancy and neonatal outcomes.

	Arcuate		Subseptate		Septate		Didelphys		Bicornuate		Unicornuate		T-shaped		Combined	
	OR (95% CI)	Stud-ies I^2(%)	OR (95% CI)	Stud-ies I^2(%)	OR (95% CI)	Stud-ies I^2(%)	OR (95% CI)	Stud-ies I^2(%)	OR (95% CI)	Stud-ies I^2(%)	OR (95% CI)	Stud-ies I^2(%)	OR (95% CI)	Stud-ies I^2(%)	OR (95% CI)	Stud-ies I^2 (%)
First trimester miscar-riage	1.21 (0.80-1.84)	3 0%	3.88 (0.86-17.54)	2 43%	1.95 (0.92-4.15)	3 63%	1.36 (0.77-2.40)	3 0%	1.56 (1.04-2.34)	4 0%	1.16 (0.56-2.39)	4 60%	N/A	--	1.62 (1.06-2.47)	7 76%
Second trimester miscarriage	0.84 (0.09-8.13)	2 58%	4.53 (1.37-15.00)	1 --	6.55 (2.66-16.16)	2 55%	1.48 (0.52-4.21)	3 0%	1.64 (0.25-10.54)	3 52%	2.07 (0.85-5.03)	4 0%	N/A	--	1.80 (1.19-2.73)	6 0%
First or second trimester miscarriage	1.14 (0.77-1.68)	4 0%	6.19 (2.30-16.66)	3 41%	2.93 (1.72-4.99)	7 49%	1.48 (0.91-2.39)	4 0%	2.09 (1.47-2.97)	6 16%	0.83 (0.45-1.56)	7 81%	5.22 (1.89-14.42)	2 0%	1.54 (1.14-2.07)	17 75%
Ectopic pregnancy	0.65 (0.15-2.83)	1 --	N/A	..	2.04 (1.03-4.04)	1 --	1.95 (0.35-10.79)	2 1%	0.65 (0.12-3.60)	3 0%	1.06 (0.32-3.51)	4 47%	5.08 (0.45-57.90)	1 --	1.30 (0.82-2.05)	6 0%
Placental abruption	15.32 (0.81-289.1)	1 --	17.45 (5.05-60.22)	1 --	10.61 (0.59-191.35)	1 --	4.45 (0.26-75.58)	1 --	12.11 (3.14-46.74)	2 81%	19.70 (1.00-387.39)	1 --	N/A	--	5.04 (3.60-7.04)	6 40%
PROM/PPROM	N/A	--	N/A	--	N/A	--	N/A	--	1.79 (1.37-2.33)	2 0%	0.46 (0.18-1.21)	1 --	N/A	--	1.71 (1.34-2.18)	9 65%
Malpresentation at delivery	11.38 (1.49-87.07)	2 41%	25.62 (10.79-60.85)	2 25%	45.48 (16.97-121.89)	2 0%	19.15 (15.16-24.18)	3 0%	17.96 (12.19-26.47)	3 27%	32.74 (6.21-172.67)	3 53%	N/A	--	21.04 (10.95-40.44)	7 97%
Preterm delivery	8.91 (3.10-25.63)	2 0%	5.24 (1.87-14.67)	2 58%	1.04* (0.51-2.01)	5 0%	4.62 (2.43-8.80)	7 74%	4.9* (3.93-6.11)	7 8%	3.85* (1.84, 8.16)	8 0%	4.45 (1.29-15.32)	1 --	4.34* (3.59-5.21)	19 56%
Premature delivery <34 weeks	N/A	--	N/A	--	16.2 (0.52-503.65)	1 --	53.78 (5.43-532.94)	1 --	11.34 (1.14-112.75)	1 --	16.20 (0.52-503.65)	1 --	N/A	--	5.36 (4.29-6.70)	6 12%
Premature delivery <32 weeks	N/A	--	N/A	--	16.2 (0.52-503.65)	1 --	6.65 (0.36-123.90)	2 81%	7.33 (0.64-83.39)	1 --	1.83 (1.00-3.35)	3 0%	N/A	--	1.64* (0.91-2,97)	6 0%
Cesarean delivery	6.44 (0.50-82.56)	2 70%	11.27 (3.01-42.23)	2 58%	5.07 (0.91-28.14)	4 82%	29.9* (8.24-126.4)	6 75%	23.8* (10.17-55.7)	6 46%	12.1* (5.64, 26.5)	6 0%	N/A	--	7.69* (4.17-14.29)	16 96%
IUGR/SGA	7.99 (0.16-405.90)	2 82%	2.54 (1.10-5.89)	2 0%	1.70 (0.43-6.78)	3 64%	3.82 (1.93-7.56)	3 36%	2.75 (1.96-3.86)	4 0%	2.74 (0.91-8.29)	4 42%	N/A	--	50* (6.11-424)	9 83%
Fetal mortality	0.93 (0.12-7.25)	2 0%	3.11 (0.35-27.44)	1 --	0.99 (0.18-5.52)	2 0%	2.67 (1.29-5.51)	3 0%	3.46 (2.00-5.99)	3 0%	2.36 (1.23-4.54)	3 0%	N/A	--	2.07 (1.56-2.73)	9 10%
Perinatal mortality	4.13 (0.47-36.57)	1 --	4.95 (0.97-25.37)	1 --	3.55 (0.83-15.08)	2 0%	6.69 (1.59-28.15)	2 25%	4.25 (1.56-11.60)	2 0%	3.05 (1.75-5.31)	3 0%	N/A	--	3.28 (2.01-5.36)	6 58%

*Adjusted OR | OR: Odds Ratio.
