# Supplementary material for: Impact of congenital uterine anomalies on obstetric and perinatal outcomes: systematic review and meta-analysis
Source: Facts Views Vis Obgyn. 2024 Mar 28;16(1):9–22. doi: 10.52054/FVVO.16.1.004 (PMC11198883; doi:10.52054/FVVO.16.1.004)
Supplement: Figure S1 — Forest plot of individual and pooled effects on ectopic pregnancy of all CUA [file FVVinObGyn-16-9-gs001.pdf]

Ectopic pregnancy (all CUA)

Study or Subgroup	Exposed Events	Exposed Total	Controls Events	Controls Total	Weight	Odds Ratio M-H, Random, 95% CI	Year
Chen 2018a	1	125	5	160	4.5%	0.25 [0.03, 2.17]	
Saravelos 2010	9	251	19	630	32.0%	1.20 [0.53, 2.68]	2010
Li 2017	7	238	20	818	27.3%	1.21 [0.50, 2.89]	2017
Kong 2021	0	75	1	241	2.0%	1.06 [0.04, 26.34]	2021
Marianna 2022	1	22	2	266	3.5%	6.29 [0.55, 72.21]	2022
Qiu 2022	7	154	38	1331	30.7%	1.62 [0.71, 3.69]	2022
**Total (95% CI)**		**865**		**3446**	**100.0%**	**1.30 [0.82, 2.05]**	
Total events	25		85				

Heterogeneity: Tau² = 0.00; Chi² = 4.28, df = 5 (P = 0.51); I² = 0%
Test for overall effect: Z = 1.12 (P = 0.26)

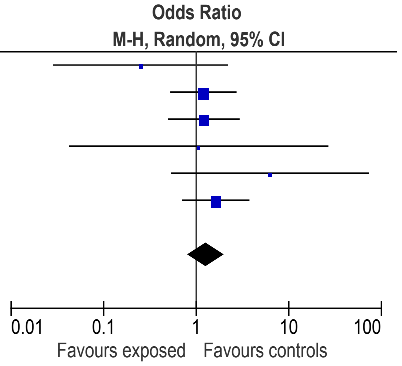

Figure S1: Forest plot of individual and pooled effects on ectopic pregnancy of all CUA
