# Supplementary material for: Impact of congenital uterine anomalies on obstetric and perinatal outcomes: systematic review and meta-analysis
Source: Facts Views Vis Obgyn. 2024 Mar 28;16(1):9–22. doi: 10.52054/FVVO.16.1.004 (PMC11198883; doi:10.52054/FVVO.16.1.004)
Supplement: Figure S2 — Forest plots of individual and pooled effects on ectopic pregnancy by type of CUA. [file FVVinObGyn-16-9-gs002.pdf]

Ectopic pregnancy by type of CUA

Study or Subgroup	Exposed Events	Exposed Total	Controls Events	Controls Total	Weight	Odds Ratio M-H, Random, 95% CI	Year
**2.4.1 Arcuate uterus**							
Saravelos 2010	2	101	19	630	100.0%	0.65 [0.15, 2.83]	2010
**Subtotal (95% CI)**		**101**		**630**	**100.0%**	**0.65 [0.15, 2.83]**	
Total events	2		19				
Heterogeneity: Not applicable							
Test for overall effect: Z = 0.57 (P = 0.57)							
**2.4.2 Septate uterus**							
Saravelos 2010	5	106	19	630	46.0%	1.59 [0.58, 4.36]	2010
Qiu 2022	6	81	23	746	54.0%	2.51 [0.99, 6.37]	2022
**Subtotal (95% CI)**		**187**		**1376**	**100.0%**	**2.04 [1.03, 4.04]**	
Total events	11		42				
Heterogeneity: Tau² = 0.00; Chi² = 0.43, df = 1 (P = 0.51); I² = 0%							
Test for overall effect: Z = 2.04 (P = 0.04)							
**2.4.3 Bicornuate uterus**							
Saravelos 2010	0	29	19	630	36.6%	0.53 [0.03, 9.02]	2010
Kong 2021	0	75	1	174	28.5%	0.77 [0.03, 19.02]	2021
Qiu 2022	0	31	6	292	34.9%	0.70 [0.04, 12.71]	2022
**Subtotal (95% CI)**		**135**		**1096**	**100.0%**	**0.65 [0.12, 3.60]**	
Total events	0		26				
Heterogeneity: Tau² = 0.00; Chi² = 0.03, df = 2 (P = 0.98); I² = 0%							
Test for overall effect: Z = 0.49 (P = 0.62)							
**2.4.4 Didelphys uterus**							
Saravelos 2010	1	10	19	630	64.7%	3.57 [0.43, 29.64]	2010
Qiu 2022	0	24	9	307	35.3%	0.64 [0.04, 11.35]	2022
**Subtotal (95% CI)**		**34**		**937**	**100.0%**	**1.95 [0.35, 10.79]**	
Total events	1		28				
Heterogeneity: Tau² = 0.01; Chi² = 1.01, df = 1 (P = 0.32); I² = 1%							
Test for overall effect: Z = 0.77 (P = 0.44)							
**2.4.5 Unicornuate uterus**							
Chen 2018a	1	125	5	160	19.5%	0.25 [0.03, 2.17]	
Saravelos 2010	1	5	19	630	18.6%	8.04 [0.86, 75.39]	2010
Li 2017	7	238	20	818	41.5%	1.21 [0.50, 2.89]	2017
Qiu 2022	1	42	9	193	20.3%	0.50 [0.06, 4.05]	2022
**Subtotal (95% CI)**		**410**		**1801**	**100.0%**	**1.06 [0.32, 3.51]**	
Total events	10		53				
Heterogeneity: Tau² = 0.70; Chi² = 5.71, df = 3 (P = 0.13); I² = 47%							
Test for overall effect: Z = 0.09 (P = 0.93)							
**2.4.6 Dysmorphic uterus**							
Marianna 2022	1	27	2	266	100.0%	5.08 [0.45, 57.90]	2022
**Subtotal (95% CI)**		**27**		**266**	**100.0%**	**5.08 [0.45, 57.90]**	
Total events	1		2				
Heterogeneity: Not applicable							
Test for overall effect: Z = 1.31 (P = 0.19)							

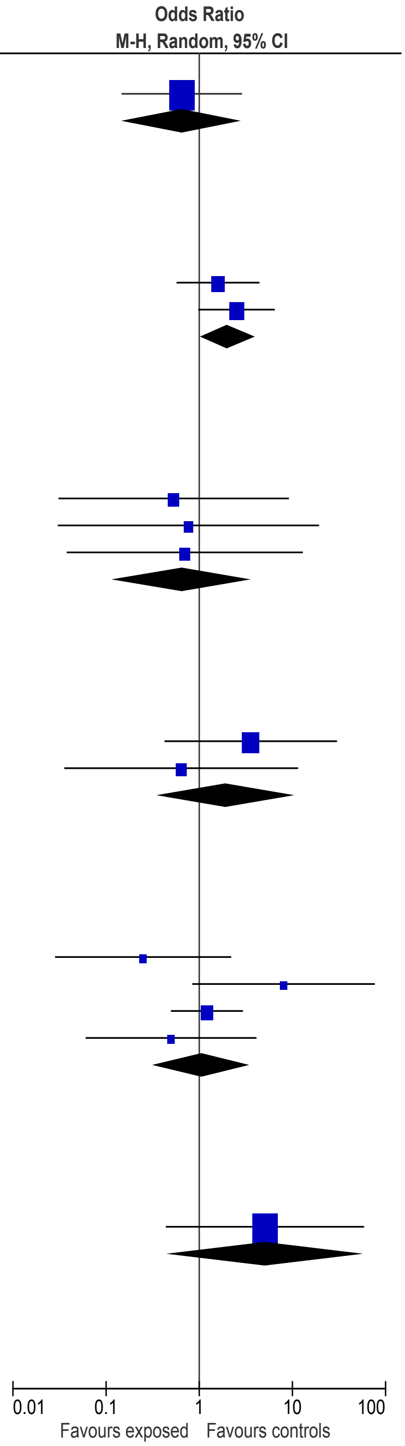

Figure S2: Forest plots of individual and pooled effects on ectopic pregnancy by type of CUA.
