# Supplementary material for: Impact of congenital uterine anomalies on obstetric and perinatal outcomes: systematic review and meta-analysis
Source: Facts Views Vis Obgyn. 2024 Mar 28;16(1):9–22. doi: 10.52054/FVVO.16.1.004 (PMC11198883; doi:10.52054/FVVO.16.1.004)
Supplement: Figure S3 — Forest plot of individual and pooled effects on placental abruption of all CUA (combined). [file FVVinObGyn-16-9-gs003.pdf]

Placental abruption (all CUA)

Study or Subgroup	Exposed Events	Exposed Total	Controls Events	Controls Total	Weight	Odds Ratio M-H, Random, 95% CI	Year
Erez 2007	5	165	65	5406	10.4%	2.57 [1.02, 6.46]	2007
Zlopasa 2007	3	105	1	182	2.1%	5.32 [0.55, 51.85]	2007
Hua 2011	4	203	400	66753	9.2%	3.33 [1.23, 9.02]	2011
Takami 2014	7	80	41	5763	12.1%	13.38 [5.81, 30.82]	2014
Cahen-Peretz 2017	35	1251	1557	255048	32.5%	4.69 [3.34, 6.58]	2017
Mastrolia 2018	39	1099	1959	279662	33.6%	5.22 [3.78, 7.20]	2018
**Total (95% CI)**		**2903**		**612814**	**100.0%**	**5.04 [3.60, 7.04]**	
Total events	93		4023				

Heterogeneity: Tau² = 0.06; Chi² = 8.30, df = 5 (P = 0.14); I² = 40%

Test for overall effect: Z = 9.45 (P < 0.00001)

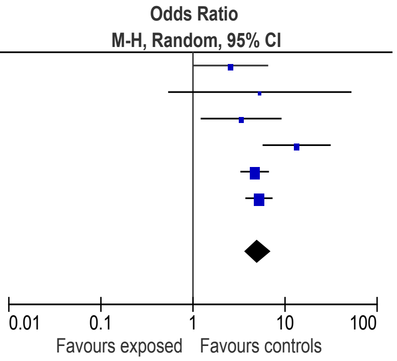

Figure S3: Forest plot of individual and pooled effects on placental abruption of all CUA (combined).
