# Supplementary material for: Impact of congenital uterine anomalies on obstetric and perinatal outcomes: systematic review and meta-analysis
Source: Facts Views Vis Obgyn. 2024 Mar 28;16(1):9–22. doi: 10.52054/FVVO.16.1.004 (PMC11198883; doi:10.52054/FVVO.16.1.004)
Supplement: Figure S4 — Forest plots of individual and pooled effects on placental abruption by type of CUA. [file FVVinObGyn-16-9-gs004.pdf]

Placental abruption by type of CUA

Study or Subgroup	Exposed Events	Exposed Total	Controls Events	Controls Total	Weight	Odds Ratio M-H, Random, 95% CI	Year
**2.6.1 Arcuate uterus**							
Takami 2014	0	4	41	5763	100.0%	15.32 [0.81, 289.14]	2014
**Subtotal (95% CI)**		**4**		**5763**	**100.0%**	**15.32 [0.81, 289.14]**	
Total events	0		41				
Heterogeneity: Not applicable							
Test for overall effect: Z = 1.82 (P = 0.07)							
**2.6.2 Subseptate uterus**							
Takami 2014	3	27	41	5763	100.0%	17.45 [5.05, 60.22]	2014
**Subtotal (95% CI)**		**27**		**5763**	**100.0%**	**17.45 [5.05, 60.22]**	
Total events	3		41				
Heterogeneity: Not applicable							
Test for overall effect: Z = 4.52 (P < 0.00001)							
**2.6.3 Septate uterus**							
Takami 2014	0	6	41	5763	100.0%	10.61 [0.59, 191.35]	2014
**Subtotal (95% CI)**		**6**		**5763**	**100.0%**	**10.61 [0.59, 191.35]**	
Total events	0		41				
Heterogeneity: Not applicable							
Test for overall effect: Z = 1.60 (P = 0.11)							
**2.6.4 Bicornuate uterus**							
Takami 2014	4	25	41	5763	43.0%	26.58 [8.74, 80.86]	2014
Mastrolia 2017	20	444	1959	279662	57.0%	6.69 [4.26, 10.49]	2017
**Subtotal (95% CI)**		**469**		**285425**	**100.0%**	**12.11 [3.14, 46.74]**	
Total events	24		2000				
Heterogeneity: $Tau^2 = 0.78$; $Chi^2 = 5.16$, df = 1 (P = 0.02); $I^2 = 81\%$							
Test for overall effect: Z = 3.62 (P = 0.0003)							
**2.6.5 Unicornuate uterus**							
Takami 2014	0	3	41	5763	100.0%	19.70 [1.00, 387.39]	2014
**Subtotal (95% CI)**		**3**		**5763**	**100.0%**	**19.70 [1.00, 387.39]**	
Total events	0		41				
Heterogeneity: Not applicable							
Test for overall effect: Z = 1.96 (P = 0.05)							
**2.6.6 Didelphys uterus**							
Takami 2014	0	15	41	5763	100.0%	4.45 [0.26, 75.58]	2014
**Subtotal (95% CI)**		**15**		**5763**	**100.0%**	**4.45 [0.26, 75.58]**	
Total events	0		41				
Heterogeneity: Not applicable							
Test for overall effect: Z = 1.03 (P = 0.30)							

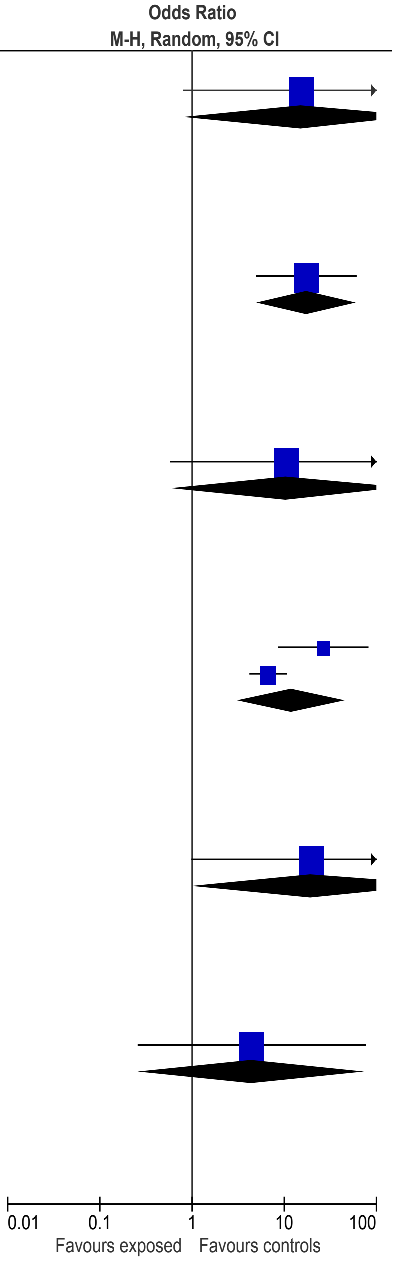

Figure S4: Forest plots of individual and pooled effects on placental abruption by type of CUA.
