# Supplementary material for: Impact of congenital uterine anomalies on obstetric and perinatal outcomes: systematic review and meta-analysis
Source: Facts Views Vis Obgyn. 2024 Mar 28;16(1):9–22. doi: 10.52054/FVVO.16.1.004 (PMC11198883; doi:10.52054/FVVO.16.1.004)
Supplement: Figure S5 — Forest plot of individual and pooled effects on PROM/PPROM of all CUA (combined). [file FVVinObGyn-16-9-gs005.pdf]

PROM/PPROM (all CUA)

Study or Subgroup	Exposed Events	Exposed Total	Controls Events	Controls Total	Weight	Odds Ratio M-H, Random, 95% CI	Year
Ben-Rafael 1991	10	237	8	381	5.2%	2.05 [0.80, 5.28]	1991
Zlopasa 2007	25	130	7	182	5.9%	5.95 [2.49, 14.24]	2007
Erez 2007	19	165	450	5406	12.2%	1.43 [0.88, 2.33]	2007
Hua 2011	14	203	1535	66753	10.9%	3.15 [1.82, 5.43]	2011
Hiersch 2016	83	243	125	486	16.6%	1.50 [1.07, 2.09]	2016
Cahen-Peretz 2017	154	1251	21993	255048	21.6%	1.49 [1.26, 1.76]	2017
Mastrolia 2018	145	1099	23170	279622	21.5%	1.68 [1.41, 2.00]	2018
Lü 2021	5	44	80	367	5.0%	0.46 [0.18, 1.21]	2021
Kong 2021	1	34	3	120	1.1%	1.18 [0.12, 11.74]	2021
**Total (95% CI)**		**3406**		**608365**	**100.0%**	**1.71 [1.34, 2.18]**	
Total events	456		47371				

Heterogeneity: Tau² = 0.06; Chi² = 22.76, df = 8 (P = 0.004); I² = 65%
Test for overall effect: Z = 4.34 (P < 0.0001)

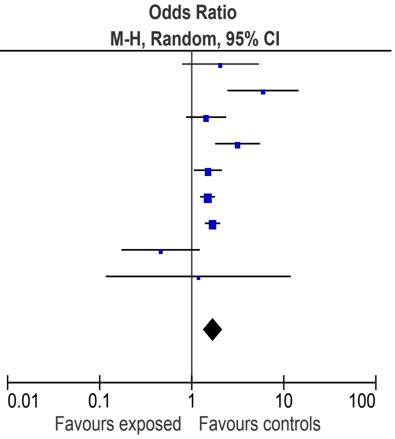

Figure S5: Forest plot of individual and pooled effects on PROM/PPROM of all CUA (combined).
