# Supplementary material for: Impact of congenital uterine anomalies on obstetric and perinatal outcomes: systematic review and meta-analysis
Source: Facts Views Vis Obgyn. 2024 Mar 28;16(1):9–22. doi: 10.52054/FVVO.16.1.004 (PMC11198883; doi:10.52054/FVVO.16.1.004)
Supplement: Figure S6 — Forest plots of individual and pooled effects on PROM/PPROM by type of CUA. [file FVVinObGyn-16-9-gs006.pdf]

PROM/PPROM by type of CUA

Study or Subgroup	Exposed Events	Exposed Total	Controls Events	Controls Total	Weight	Odds Ratio M-H, Random, 95% CI	Year
**2.7.4 Bicornuate uterus**							
Mastrolia 2017	62	444	23170	279662	98.6%	1.80 [1.37, 2.35]	2017
Kong 2021	1	34	3	120	1.4%	1.18 [0.12, 11.74]	2021
**Subtotal (95% CI)**		**478**		**279782**	**100.0%**	**1.79 [1.37, 2.33]**	
Total events	63		23173				
Heterogeneity: Tau² = 0.00; Chi² = 0.13, df = 1 (P = 0.72); I² = 0%							
Test for overall effect: Z = 4.26 (P < 0.0001)							
**2.7.6 Unicornuate uterus**							
Lü 2021	5	44	80	367	100.0%	0.46 [0.18, 1.21]	2021
**Subtotal (95% CI)**		**44**		**367**	**100.0%**	**0.46 [0.18, 1.21]**	
Total events	5		80				
Heterogeneity: Not applicable							
Test for overall effect: Z = 1.58 (P = 0.11)							

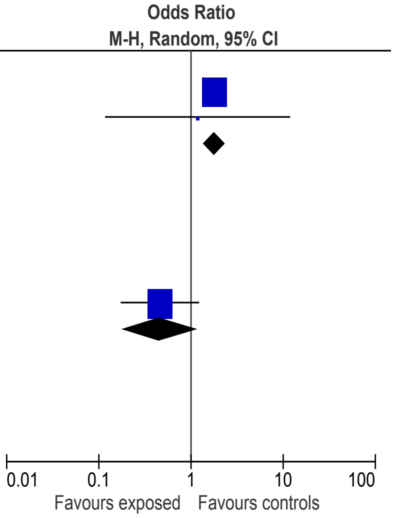

Figure S6: Forest plots of individual and pooled effects on PROM/PPROM by type of CUA.
