# Supplementary material for: Impact of congenital uterine anomalies on obstetric and perinatal outcomes: systematic review and meta-analysis
Source: Facts Views Vis Obgyn. 2024 Mar 28;16(1):9–22. doi: 10.52054/FVVO.16.1.004 (PMC11198883; doi:10.52054/FVVO.16.1.004)
Supplement: Figure S7 — Forest plot of individual and pooled effects on fetal malpresentation at delivery of all CUA (combined). [file FVVinObGyn-16-9-gs007.pdf]

Fetal malpresentation at delivery (all CUA)

Study or Subgroup	Exposed Events	Exposed Total	Controls Events	Controls Total	Weight	Odds Ratio M-H, Random, 95% CI	Year
Ben-Rafael 1991	29	88	3	141	10.4%	22.61 [6.63, 77.13]	1991
Zlopasa 2007	53	105	6	182	12.6%	29.90 [12.17, 73.47]	2007
Hua 2011	48	203	2002	66753	15.8%	10.02 [7.22, 13.89]	2011
Takami 2014	31	80	228	5763	15.2%	15.36 [9.61, 24.55]	2014
Cahen-Peretz 2017	1027	1251	16552	255048	16.3%	66.06 [57.12, 76.40]	2017
Mastrolia 2018	547	1099	12923	279622	16.4%	20.45 [18.15, 23.05]	2018
Lü 2021	16	44	16	367	13.3%	12.54 [5.67, 27.69]	2021
**Total (95% CI)**		**2870**		**607876**	**100.0%**	**21.04 [10.95, 40.44]**	
Total events	1751		31730				

Heterogeneity: Tau² = 0.67; Chi² = 215.44, df = 6 (P < 0.00001); I² = 97%
Test for overall effect: Z = 9.14 (P < 0.00001)

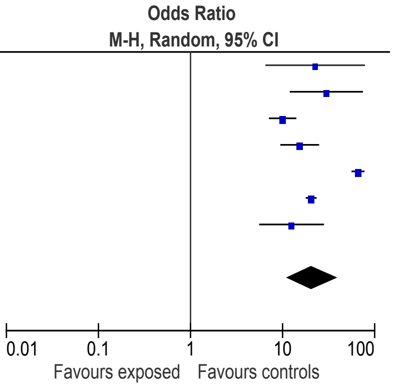

Figure S7: Forest plot of individual and pooled effects on fetal malpresentation at delivery of all CUA (combined).
