# Supplementary material for: Impact of congenital uterine anomalies on obstetric and perinatal outcomes: systematic review and meta-analysis
Source: Facts Views Vis Obgyn. 2024 Mar 28;16(1):9–22. doi: 10.52054/FVVO.16.1.004 (PMC11198883; doi:10.52054/FVVO.16.1.004)
Supplement: Figure S8 — Forest plots of individual and pooled effects on fetal malpresentation at delivery by type of CUA. [file FVVinObGyn-16-9-gs008.pdf]

Fetal malpresentation at delivery by type of CUA

Study or Subgroup	Exposed Events	Exposed Total	Controls Events	Controls Total	Weight	Odds Ratio M-H, Random, 95% CI	Year
**2.8.1 Arcuate uterus**							
Zlopasa 2007	4	9	6	182	66.6%	23.47 [5.00, 110.14]	2007
Takami 2014	0	4	228	5763	33.4%	2.69 [0.14, 50.14]	2014
**Subtotal (95% CI)**		**13**		**5945**	**100.0%**	**11.38 [1.49, 87.07]**	
Total events	4		234				
Heterogeneity: Tau² = 1.00; Chi² = 1.70, df = 1 (P = 0.19); I² = 41%							
Test for overall effect: Z = 2.34 (P = 0.02)							
**2.8.2 Subseptate uterus**							
Zlopasa 2007	7	11	6	182	28.5%	51.33 [11.76, 224.04]	2007
Takami 2014	12	27	228	5763	71.5%	19.42 [8.99, 41.97]	2014
**Subtotal (95% CI)**		**38**		**5945**	**100.0%**	**25.62 [10.79, 60.85]**	
Total events	19		234				
Heterogeneity: Tau² = 0.12; Chi² = 1.33, df = 1 (P = 0.25); I² = 25%							
Test for overall effect: Z = 7.35 (P < 0.00001)							
**2.8.3 Septate uterus**							
Zlopasa 2007	12	20	6	182	66.5%	44.00 [13.13, 147.45]	2007
Takami 2014	4	6	228	5763	33.5%	48.55 [8.85, 266.45]	2014
**Subtotal (95% CI)**		**26**		**5945**	**100.0%**	**45.48 [16.97, 121.89]**	
Total events	16		234				
Heterogeneity: Tau² = 0.00; Chi² = 0.01, df = 1 (P = 0.93); I² = 0%							
Test for overall effect: Z = 7.59 (P < 0.00001)							
**2.8.4 Bicornuate uterus**							
Zlopasa 2007	17	38	6	182	12.1%	23.75 [8.44, 66.84]	2007
Takami 2014	7	25	228	5763	15.8%	9.44 [3.90, 22.83]	2014
Mastrolia 2017	217	444	12923	279662	72.1%	19.73 [16.37, 23.79]	2017
**Subtotal (95% CI)**		**507**		**285607**	**100.0%**	**17.96 [12.19, 26.47]**	
Total events	241		13157				
Heterogeneity: Tau² = 0.05; Chi² = 2.73, df = 2 (P = 0.26); I² = 27%							
Test for overall effect: Z = 14.60 (P < 0.00001)							
**2.8.5 Unicornuate uterus**							
Zlopasa 2007	4	4	6	182	20.1%	244.38 [11.87, 5031.78]	2007
Takami 2014	2	3	228	5763	26.7%	48.55 [4.39, 537.41]	2014
Lü 2021	16	44	16	367	53.1%	12.54 [5.67, 27.69]	2021
**Subtotal (95% CI)**		**51**		**6312**	**100.0%**	**32.74 [6.21, 172.67]**	
Total events	22		250				
Heterogeneity: Tau² = 1.19; Chi² = 4.29, df = 2 (P = 0.12); I² = 53%							
Test for overall effect: Z = 4.11 (P < 0.0001)							
**2.8.6 Didelphys uterus**							
Zlopasa 2007	9	23	6	182	4.0%	18.86 [5.87, 60.61]	2007
Takami 2014	6	15	228	5763	5.0%	16.18 [5.71, 45.86]	2014
Cahen-Peretz 2017	128	258	12357	255048	91.0%	19.34 [15.14, 24.70]	2017
**Subtotal (95% CI)**		**296**		**260993**	**100.0%**	**19.15 [15.16, 24.18]**	
Total events	143		12591				
Heterogeneity: Tau² = 0.00; Chi² = 0.11, df = 2 (P = 0.95); I² = 0%							
Test for overall effect: Z = 24.79 (P < 0.00001)							

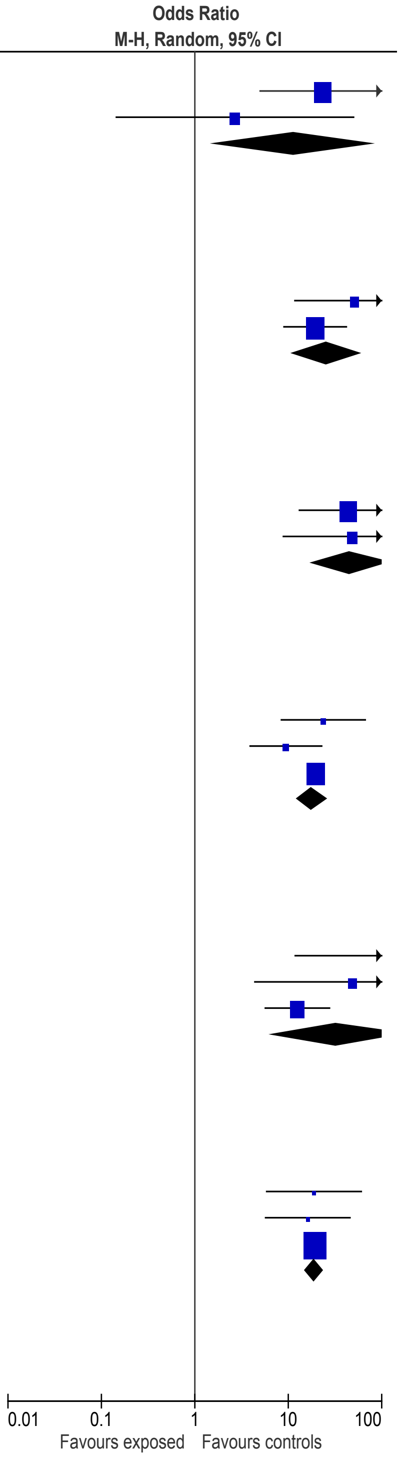

Figure S8: Forest plots of individual and pooled effects on fetal malpresentation at delivery by type of CUA.
