# Supplementary material for: Impact of congenital uterine anomalies on obstetric and perinatal outcomes: systematic review and meta-analysis
Source: Facts Views Vis Obgyn. 2024 Mar 28;16(1):9–22. doi: 10.52054/FVVO.16.1.004 (PMC11198883; doi:10.52054/FVVO.16.1.004)
Supplement: Figure S9 — Forest plots of individual and pooled effects on preterm delivery (A) preterm delivery < 34 wweks (B) and preterm delivery < 32 weeks of all CUA (combined). [file FVVinObGyn-16-9-gs009.pdf]

Preterm delivery (all CUA)

A) Preterm birth

Study or Subgroup	Exposed Events	Exposed Total	Controls Events	Controls Total	Weight	Odds Ratio M-H, Random, 95% CI	Year
Chen 2018a	37	122	32	149	5.7%	1.59 [0.92, 2.76]	
Ben-Rafael 1991	29	245	29	413	5.8%	1.78 [1.03, 3.05]	1991
Crane 1992	14	52	4	122	3.2%	10.87 [3.37, 35.01]	1992
Leible 1998	2	14	1	30	1.1%	4.83 [0.40, 58.47]	1998
Zlopasa 2007	50	246	23	379	5.9%	3.95 [2.34, 6.67]	2007
Erez 2007	33	165	595	5406	6.4%	2.02 [1.37, 2.99]	2007
Hua 2011	80	203	6942	66753	6.8%	5.60 [4.22, 7.43]	2011
Takami 2014	17	80	276	5763	5.7%	5.36 [3.10, 9.29]	2014
Hiersch 2016	61	243	30	486	6.1%	5.09 [3.19, 8.15]	2016
Li 2017	57	238	123	818	6.6%	1.78 [1.25, 2.54]	2017
Cahen-Peretz 2017	332	1251	18516	255048	7.2%	4.61 [4.07, 5.24]	2017
Prior 2018	33	152	102	722	6.2%	1.69 [1.09, 2.61]	2018
Mastrolia 2018	287	1099	20682	279662	7.2%	4.43 [3.87, 5.07]	2018
Ouyang 2020	58	206	98	314	6.4%	0.86 [0.59, 1.27]	2020
Cai 2021	12	65	9	195	4.1%	4.68 [1.87, 11.70]	2021
Lü 2021	9	44	22	367	4.4%	4.03 [1.72, 9.43]	2021
Kong 2021	3	35	11	112	2.8%	0.86 [0.23, 3.28]	2021
Marianna 2022	2	22	10	266	2.2%	2.56 [0.52, 12.49]	2022
Qiu 2022	22	154	160	1331	6.0%	1.22 [0.75, 1.97]	2022
**Total (95% CI)**		**4636**		**618336**	**100.0%**	**2.79 [2.10, 3.70]**	
Total events	1138		47665				

Heterogeneity: Tau² = 0.28; Chi² = 173.74, df = 18 (P < 0.00001); I² = 90%
Test for overall effect: Z = 7.12 (P < 0.00001)

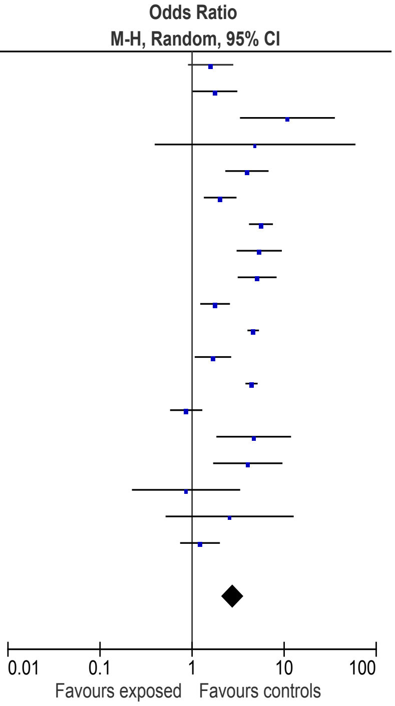

B) Preterm birth < 34 weeks

Study or Subgroup	Exposed Events	Exposed Total	Controls Events	Controls Total	Weight	Odds Ratio M-H, Random, 95% CI	Year
Crane 1992	7	52	1	122	1.1%	18.82 [2.25, 157.29]	1992
Cooney 1998	5	17	2	62	1.6%	12.50 [2.17, 72.15]	1998
Hua 2011	29	203	1735	66753	24.5%	6.25 [4.20, 9.28]	2011
Hiersch 2016	22	243	7	486	6.2%	6.81 [2.87, 16.18]	2016
Cahen-Peretz 2017	106	1351	4199	255048	58.2%	5.09 [4.16, 6.22]	2017
Prior 2018	12	152	20	722	8.4%	3.01 [1.44, 6.30]	2018
**Total (95% CI)**		**2018**		**323193**	**100.0%**	**5.36 [4.29, 6.70]**	
Total events	181		5964				

Heterogeneity: Tau² = 0.01; Chi² = 5.71, df = 5 (P = 0.34); I² = 12%
Test for overall effect: Z = 14.81 (P < 0.00001)

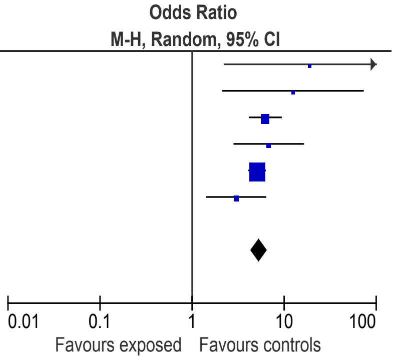

C) Preterm birth < 32 weeks

Study or Subgroup	Exposed Events	Exposed Total	Controls Events	Controls Total	Weight	Odds Ratio M-H, Random, 95% CI	Year
Chen 2018a	6	122	4	129	15.6%	1.62 [0.44, 5.87]	
Crane 1992	5	52	1	122	6.9%	12.87 [1.46, 113.11]	1992
Hiersch 2016	10	243	3	486	15.4%	6.91 [1.88, 25.35]	2016
Li 2017	12	187	27	709	29.7%	1.73 [0.86, 3.49]	2017
Prior 2018	11	152	13	722	26.0%	4.25 [1.87, 9.69]	2018
Cai 2021	1	51	3	159	6.4%	1.04 [0.11, 10.22]	2021
**Total (95% CI)**		**807**		**2327**	**100.0%**	**2.98 [1.60, 5.54]**	
Total events	45		51				

Heterogeneity: Tau² = 0.21; Chi² = 7.98, df = 5 (P = 0.16); I² = 37%
Test for overall effect: Z = 3.45 (P = 0.0006)

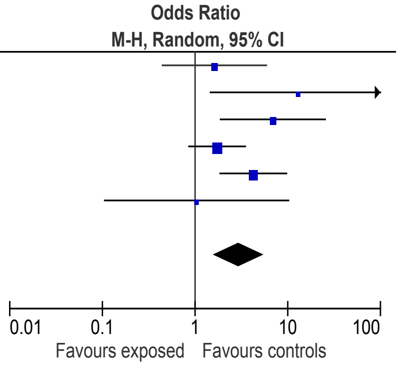

Figure S9: Forest plots of individual and pooled effects on preterm delivery (A) preterm delivery < 34 wweks (B) and preterm delivery < 32 weeks of all CUA (combined).
