# Supplementary material for: Impact of congenital uterine anomalies on obstetric and perinatal outcomes: systematic review and meta-analysis
Source: Facts Views Vis Obgyn. 2024 Mar 28;16(1):9–22. doi: 10.52054/FVVO.16.1.004 (PMC11198883; doi:10.52054/FVVO.16.1.004)
Supplement: Figure S10 — Forest plots of individual and pooled effects on preterm delivery by type of CUA. [file FVVinObGyn-16-9-gs010.pdf]

Preterm delivery by type of CUA
Preterm delivery

Study or Subgroup	Exposed Events	Exposed Total	Controls Events	Controls Total	Weight	Odds Ratio M-H, Random, 95% CI	Year
**2.9.1 Arcuate uterus**							
Zlopasa 2007	5	13	23	379	78.3%	9.67 [2.93, 31.94]	2007
Takami 2014	1	4	276	5763	21.7%	6.63 [0.69, 63.91]	2014
**Subtotal (95% CI)**		**17**		**6142**	**100.0%**	**8.91 [3.10, 25.63]**	
Total events	6		299				
Heterogeneity: Tau² = 0.00; Chi² = 0.08, df = 1 (P = 0.77); I² = 0%							
Test for overall effect: Z = 4.06 (P < 0.0001)							
**2.9.2 Subseptate uterus**							
Zlopasa 2007	5	31	23	379	45.4%	2.98 [1.05, 8.47]	2007
Takami 2014	8	27	276	5763	54.6%	8.37 [3.63, 19.29]	2014
**Subtotal (95% CI)**		**58**		**6142**	**100.0%**	**5.24 [1.87, 14.67]**	
Total events	13		299				
Heterogeneity: Tau² = 0.32; Chi² = 2.39, df = 1 (P = 0.12); I² = 58%							
Test for overall effect: Z = 3.15 (P = 0.002)							
**2.9.3 Septate uterus**							
Crane 1992	0	2	4	122	5.0%	5.27 [0.22, 126.51]	1992
Zlopasa 2007	8	60	23	379	28.0%	2.38 [1.01, 5.60]	2007
Takami 2014	0	6	276	5763	6.0%	1.53 [0.09, 27.17]	2014
Hiersch 2016	9	38	30	486	28.5%	4.72 [2.05, 10.86]	2016
Qiu 2022	11	81	99	746	32.5%	1.03 [0.53, 2.01]	2022
**Subtotal (95% CI)**		**187**		**7496**	**100.0%**	**2.23 [1.05, 4.75]**	
Total events	28		432				
Heterogeneity: Tau² = 0.34; Chi² = 8.65, df = 4 (P = 0.07); I² = 54%							
Test for overall effect: Z = 2.08 (P = 0.04)							
**2.9.4 Bicornuate uterus**							
Crane 1992	7	35	4	122	6.5%	7.38 [2.02, 26.95]	1992
Zlopasa 2007	14	91	23	379	15.0%	2.81 [1.39, 5.72]	2007
Takami 2014	6	25	276	5763	10.8%	6.28 [2.49, 15.84]	2014
Hiersch 2016	35	156	30	486	19.9%	4.40 [2.60, 7.45]	2016
Mastrolia 2017	138	444	22066	279662	30.6%	5.26 [4.30, 6.44]	2017
Kong 2021	3	35	11	112	6.2%	0.86 [0.23, 3.28]	2021
Qiu 2022	7	31	39	392	11.1%	2.64 [1.07, 6.52]	2022
**Subtotal (95% CI)**		**817**		**286916**	**100.0%**	**3.99 [2.76, 5.76]**	
Total events	210		22449				
Heterogeneity: Tau² = 0.10; Chi² = 12.12, df = 6 (P = 0.06); I² = 50%							
Test for overall effect: Z = 7.37 (P < 0.00001)							
**2.9.5 Unicornuate uterus**							
Chen 2018a	37	122	32	149	19.7%	1.59 [0.92, 2.76]	
Crane 1992	1	2	4	122	3.0%	29.50 [1.55, 561.08]	1992
Zlopasa 2007	3	8	23	379	8.7%	9.29 [2.09, 41.30]	2007
Takami 2014	1	3	276	5763	4.3%	9.94 [0.90, 109.96]	2014
Hiersch 2016	7	27	30	486	14.3%	5.32 [2.09, 13.57]	2016
Li 2017	57	238	123	818	22.3%	1.78 [1.25, 2.54]	2017
Lü 2021	9	44	22	367	15.5%	4.03 [1.72, 9.43]	2021
Qiu 2022	4	42	22	193	12.1%	0.82 [0.27, 2.51]	2022
**Subtotal (95% CI)**		**486**		**8277**	**100.0%**	**2.85 [1.65, 4.93]**	
Total events	119		532				
Heterogeneity: Tau² = 0.32; Chi² = 19.48, df = 7 (P = 0.007); I² = 64%							
Test for overall effect: Z = 3.74 (P = 0.0002)							
**2.9.6 Didelphys uterus**							
Crane 1992	6	13	4	122	10.4%	25.29 [5.77, 110.76]	1992
Zlopasa 2007	3	8	23	379	10.3%	9.29 [2.09, 41.30]	2007
Takami 2014	1	15	276	5763	7.0%	1.42 [0.19, 10.84]	2014
Hiersch 2016	9	22	30	486	15.7%	10.52 [4.17, 26.58]	2016
Cahen-Peretz 2017	72	258	18516	255048	22.3%	4.94 [3.77, 6.49]	2017
Cai 2021	25	83	28	249	19.2%	3.40 [1.84, 6.27]	2021
Qiu 2022	7	24	34	107	15.2%	0.88 [0.34, 2.33]	2022
**Subtotal (95% CI)**		**423**		**262154**	**100.0%**	**4.62 [2.43, 8.80]**	
Total events	123		18911				
Heterogeneity: Tau² = 0.46; Chi² = 23.33, df = 6 (P = 0.0007); I² = 74%							
Test for overall effect: Z = 4.67 (P < 0.00001)							
**2.9.7 Dysmorphic uterus**							
Marianna 2022	4	27	10	266	100.0%	4.45 [1.29, 15.32]	2022
**Subtotal (95% CI)**		**27**		**266**	**100.0%**	**4.45 [1.29, 15.32]**	
Total events	4		10				
Heterogeneity: Not applicable							
Test for overall effect: Z = 2.37 (P = 0.02)							

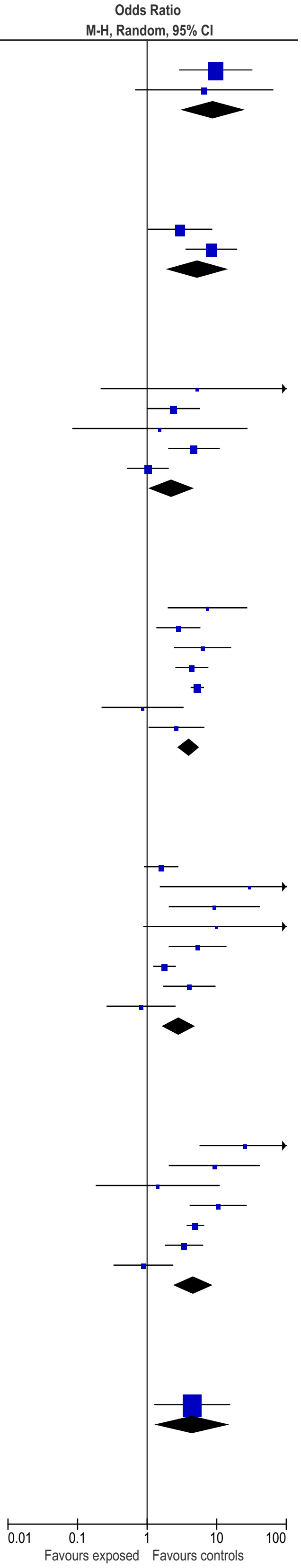

Figure S10: Forest plots of individual and pooled effects on preterm delivery by type of CUA.
