# Supplementary material for: Impact of congenital uterine anomalies on obstetric and perinatal outcomes: systematic review and meta-analysis
Source: Facts Views Vis Obgyn. 2024 Mar 28;16(1):9–22. doi: 10.52054/FVVO.16.1.004 (PMC11198883; doi:10.52054/FVVO.16.1.004)
Supplement: Figure S11 — Forest plots of individual and pooled effects on preterm delivery < 34 weeks by type of CUA. [file FVVinObGyn-16-9-gs011.pdf]

Preterm delivery < 34 weeks

Study or Subgroup	Exposed Events	Exposed Total	Controls Events	Controls Total	Weight	Odds Ratio M-H, Random, 95% CI
**2.10.3 Septate uterus**						
Crane 1992	0	2	1	122	100.0%	16.20 [0.52, 503.65]
**Subtotal (95% CI)**		**2**		**122**	**100.0%**	**16.20 [0.52, 503.65]**
Total events	0		1			
Heterogeneity: Not applicable						
Test for overall effect: Z = 1.59 (P = 0.11)						
**2.10.4 Bicornuate uterus**						
Crane 1992	3	35	1	122	100.0%	11.34 [1.14, 112.75]
**Subtotal (95% CI)**		**35**		**122**	**100.0%**	**11.34 [1.14, 112.75]**
Total events	3		1			
Heterogeneity: Not applicable						
Test for overall effect: Z = 2.07 (P = 0.04)						
**2.10.5 Unicornuate uterus**						
Crane 1992	0	2	1	122	100.0%	16.20 [0.52, 503.65]
**Subtotal (95% CI)**		**2**		**122**	**100.0%**	**16.20 [0.52, 503.65]**
Total events	0		1			
Heterogeneity: Not applicable						
Test for overall effect: Z = 1.59 (P = 0.11)						
**2.10.6 Didelphys uterus**						
Crane 1992	4	13	1	122	100.0%	53.78 [5.43, 532.94]
**Subtotal (95% CI)**		**13**		**122**	**100.0%**	**53.78 [5.43, 532.94]**
Total events	4		1			
Heterogeneity: Not applicable						
Test for overall effect: Z = 3.41 (P = 0.0007)						

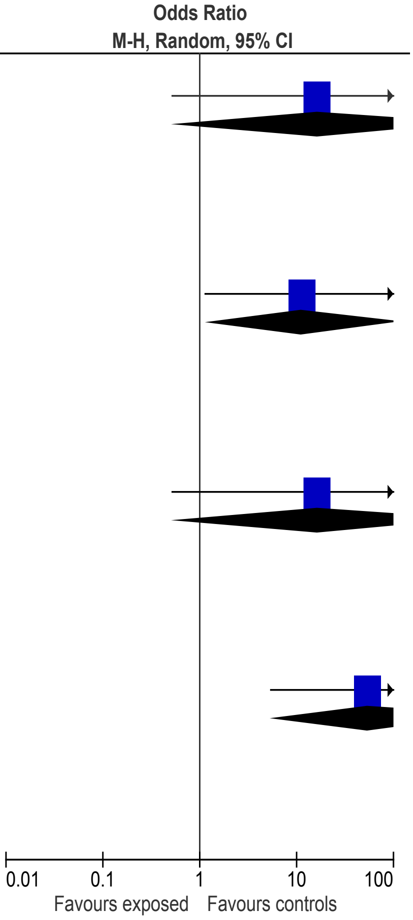

Figure S11: Forest plots of individual and pooled effects on preterm delivery < 34 weeks by type of CUA.
