# Supplementary material for: Impact of congenital uterine anomalies on obstetric and perinatal outcomes: systematic review and meta-analysis
Source: Facts Views Vis Obgyn. 2024 Mar 28;16(1):9–22. doi: 10.52054/FVVO.16.1.004 (PMC11198883; doi:10.52054/FVVO.16.1.004)
Supplement: Figure S12 — Forest plots of individual and pooled effects on preterm delivery < 32 weeks by type of CUA. [file FVVinObGyn-16-9-gs012.pdf]

Preterm delivery < 32 weeks

Study or Subgroup	Exposed Events	Exposed Total	Controls Events	Controls Total	Weight	Odds Ratio M-H, Random, 95% CI	Year
**2.11.3 Septate uterus**							
Crane 1992	0	2	1	122	100.0%	16.20 [0.52, 503.65]	1992
**Subtotal (95% CI)**		**2**		**122**	**100.0%**	**16.20 [0.52, 503.65]**	
Total events	0		1				
Heterogeneity: Not applicable							
Test for overall effect: Z = 1.59 (P = 0.11)							
**2.11.4 Bicornuate uterus**							
Crane 1992	2	35	1	122	100.0%	7.33 [0.64, 83.39]	1992
**Subtotal (95% CI)**		**35**		**122**	**100.0%**	**7.33 [0.64, 83.39]**	
Total events	2		1				
Heterogeneity: Not applicable							
Test for overall effect: Z = 1.61 (P = 0.11)							
**2.11.5 Unicornuate uterus**							
Chen 2018a	6	122	4	129	22.0%	1.62 [0.44, 5.87]	
Crane 1992	0	2	1	122	3.1%	16.20 [0.52, 503.65]	1992
Li 2017	12	187	27	709	74.9%	1.73 [0.86, 3.49]	2017
**Subtotal (95% CI)**		**311**		**960**	**100.0%**	**1.83 [1.00, 3.35]**	
Total events	18		32				
Heterogeneity: Tau² = 0.00; Chi² = 1.62, df = 2 (P = 0.44); I² = 0%							
Test for overall effect: Z = 1.95 (P = 0.05)							
**2.11.6 Didelphys uterus**							
Crane 1992	3	13	1	122	43.6%	36.30 [3.45, 381.87]	1992
Cai 2021	6	65	11	205	56.4%	1.79 [0.64, 5.06]	2021
**Subtotal (95% CI)**		**78**		**327**	**100.0%**	**6.65 [0.36, 123.90]**	
Total events	9		12				
Heterogeneity: Tau² = 3.67; Chi² = 5.26, df = 1 (P = 0.02); I² = 81%							
Test for overall effect: Z = 1.27 (P = 0.20)							

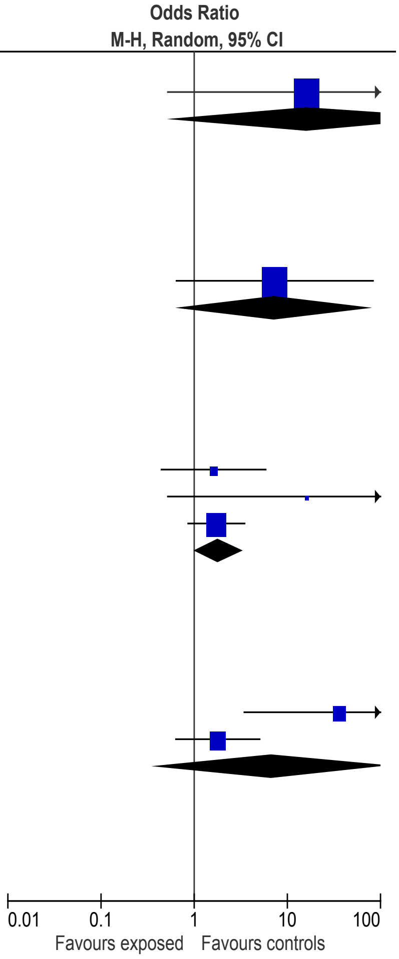

Figure S12: Forest plots of individual and pooled effects on preterm delivery < 32 weeks by type of CUA.
