# Supplementary material for: Impact of congenital uterine anomalies on obstetric and perinatal outcomes: systematic review and meta-analysis
Source: Facts Views Vis Obgyn. 2024 Mar 28;16(1):9–22. doi: 10.52054/FVVO.16.1.004 (PMC11198883; doi:10.52054/FVVO.16.1.004)
Supplement: Figure S13 — Forest plot of individual and pooled effects on cesarean delivery of all CUA (combined). [file FVVinObGyn-16-9-gs013.pdf]

Cesarean delivery (all CUA)

Study or Subgroup	Exposed Events	Exposed Total	Controls Events	Controls Total	Weight	Odds Ratio M-H, Random, 95% CI	Year
Crane 1992	32	52	31	122	6.1%	4.70 [2.35, 9.38]	1992
Cooney 1998	8	17	19	62	5.6%	2.01 [0.67, 6.01]	1998
Erez 2007	103	165	2666	5406	6.4%	1.71 [1.24, 2.35]	2007
Zlopasa 2007	78	105	26	182	6.2%	17.33 [9.48, 31.69]	2007
Hua 2011	70	203	10814	66753	6.5%	2.72 [2.04, 3.64]	2011
Takami 2014	50	80	1025	5763	6.4%	7.70 [4.87, 12.18]	2014
Hiersch 2016	200	243	108	486	6.4%	16.28 [10.99, 24.11]	2016
Li 2017	155	187	557	709	6.4%	1.32 [0.87, 2.01]	2017
Cahen-Peretz 2017	975	1251	34739	255048	6.5%	22.40 [19.59, 25.62]	2017
Mastrolia 2017	346	444	38740	279662		Not estimable	2017
Mastrolia 2018	881	1099	38740	279622	6.5%	25.13 [21.66, 29.16]	2018
Prior 2018	66	152	291	722	6.4%	1.14 [0.80, 1.62]	2018
Ouyang 2020	168	206	268	314	6.3%	0.76 [0.47, 1.22]	2020
Kong 2021	26	35	52	112	5.9%	3.33 [1.43, 7.75]	2021
Lü 2021	38	44	134	367	5.9%	11.01 [4.54, 26.73]	2021
Cai 2021	52	65	146	205	6.1%	1.62 [0.82, 3.19]	2021
Qiu 2022	97	112	846	1100	6.3%	1.94 [1.11, 3.40]	2022
**Total (95% CI)**		**4016**		**616973**	**100.0%**	**4.15 [2.12, 8.15]**	
Total events	2999		90462				

Heterogeneity: Tau² = 1.81; Chi² = 931.70, df = 15 (P < 0.00001); I² = 98%

Test for overall effect: Z = 4.14 (P < 0.0001)

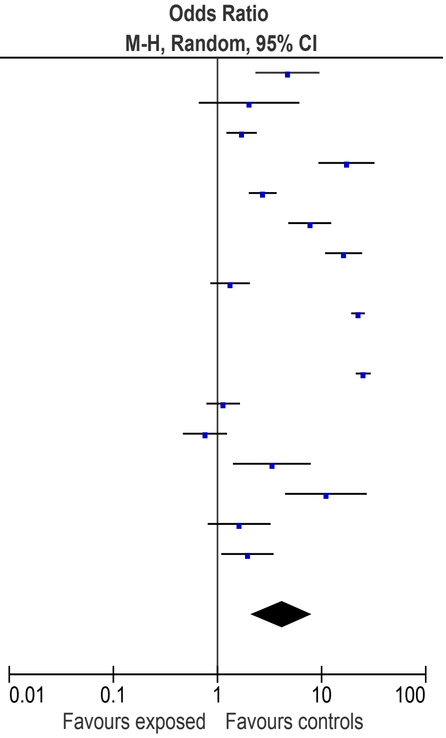

Figure S13: Forest plot of individual and pooled effects on cesarean delivery of all CUA (combined).
