# Supplementary material for: Impact of congenital uterine anomalies on obstetric and perinatal outcomes: systematic review and meta-analysis
Source: Facts Views Vis Obgyn. 2024 Mar 28;16(1):9–22. doi: 10.52054/FVVO.16.1.004 (PMC11198883; doi:10.52054/FVVO.16.1.004)
Supplement: Figure S14 — Forest plots of individual and pooled effects on cesarean delivery by type of CUA. [file FVVinObGyn-16-9-gs014.pdf]

Cesarean delivery by type of CUA

Study or Subgroup	Exposed Events	Exposed Total	Controls Events	Controls Total	Weight	Odds Ratio M-H, Random, 95% CI	Year
**2.12.1 Arcuate uterus**							
Zlopasa 2007	7	9	26	182	54.7%	21.00 [4.13, 106.69]	2007
Takami 2014	1	4	1025	5763	45.3%	1.54 [0.16, 14.83]	2014
**Subtotal (95% CI)**		**13**		**5945**	**100.0%**	**6.44 [0.50, 82.56]**	
Total events	8		1051				
Heterogeneity: Tau² = 2.41; Chi² = 3.38, df = 1 (P = 0.07); I² = 70%							
Test for overall effect: Z = 1.43 (P = 0.15)							
**2.12.2 Subseptate uterus**							
Zlopasa 2007	9	11	26	182	37.1%	27.00 [5.52, 132.06]	2007
Takami 2014	16	27	1025	5763	62.9%	6.72 [3.11, 14.53]	2014
**Subtotal (95% CI)**		**38**		**5945**	**100.0%**	**11.27 [3.01, 42.23]**	
Total events	25		1051				
Heterogeneity: Tau² = 0.57; Chi² = 2.40, df = 1 (P = 0.12); I² = 58%							
Test for overall effect: Z = 3.59 (P = 0.0003)							
**2.12.3 Septate uterus**							
Crane 1992	0	2	31	122	16.3%	0.58 [0.03, 12.43]	1992
Zlopasa 2007	15	20	26	182	29.8%	18.00 [6.03, 53.75]	2007
Takami 2014	5	6	1025	5763	22.1%	23.11 [2.70, 198.04]	2014
Qiu 2022	48	56	497	632	31.8%	1.63 [0.75, 3.53]	2022
**Subtotal (95% CI)**		**84**		**6699**	**100.0%**	**5.07 [0.91, 28.14]**	
Total events	68		1579				
Heterogeneity: Tau² = 2.25; Chi² = 16.64, df = 3 (P = 0.0008); I² = 82%							
Test for overall effect: Z = 1.86 (P = 0.06)							
**2.12.4 Bicornuate uterus**							
Crane 1992	21	35	31	122	18.0%	4.40 [2.00, 9.70]	1992
Zlopasa 2007	26	38	26	182	18.0%	13.00 [5.84, 28.94]	2007
Takami 2014	12	25	1025	5763	18.0%	4.27 [1.94, 9.38]	2014
Mastrolia 2017	346	444	38740	279662	19.8%	21.96 [17.54, 27.48]	2017
Kong 2021	26	35	82	112	17.7%	1.06 [0.44, 2.51]	2021
Qiu 2022	22	22	244	315	8.5%	13.16 [0.79, 219.63]	2022
**Subtotal (95% CI)**		**599**		**286156**	**100.0%**	**6.23 [2.12, 18.33]**	
Total events	453		40148				
Heterogeneity: Tau² = 1.52; Chi² = 66.99, df = 5 (P < 0.00001); I² = 93%							
Test for overall effect: Z = 3.32 (P = 0.0009)							
**2.12.5 Unicornuate uterus**							
Crane 1992	2	2	31	122	8.8%	14.52 [0.68, 310.76]	1992
Zlopasa 2007	4	4	26	182	9.3%	53.15 [2.78, 1016.12]	2007
Takami 2014	2	3	1025	5763	11.8%	9.24 [0.84, 102.05]	2014
Li 2017	155	187	557	709	25.2%	1.32 [0.87, 2.01]	2017
Lü 2021	38	44	134	367	22.5%	11.01 [4.54, 26.73]	2021
Qiu 2022	27	34	105	153	22.4%	1.76 [0.72, 4.33]	2022
**Subtotal (95% CI)**		**274**		**7296**	**100.0%**	**4.97 [1.63, 15.18]**	
Total events	228		1878				
Heterogeneity: Tau² = 1.24; Chi² = 26.19, df = 5 (P < 0.0001); I² = 81%							
Test for overall effect: Z = 2.81 (P = 0.005)							
**2.12.6 Didelphys uterus**							
Crane 1992	9	13	31	122	17.4%	6.60 [1.90, 22.97]	1992
Zlopasa 2007	17	23	26	182	18.3%	17.00 [6.14, 47.10]	2007
Takami 2014	14	15	1025	5763	14.1%	64.71 [8.50, 492.68]	2014
Cahen-Peretz 2017	216	258	34739	255048	20.1%	32.62 [23.43, 45.40]	2017
Cai 2021	52	65	146	205	19.3%	1.62 [0.82, 3.19]	2021
Qiu 2022	18	18	193	251	10.9%	11.19 [0.66, 188.46]	2022
**Subtotal (95% CI)**		**392**		**261571**	**100.0%**	**12.02 [3.04, 47.50]**	
Total events	326		36160				
Heterogeneity: Tau² = 2.42; Chi² = 65.81, df = 5 (P < 0.00001); I² = 92%							
Test for overall effect: Z = 3.55 (P = 0.0004)							

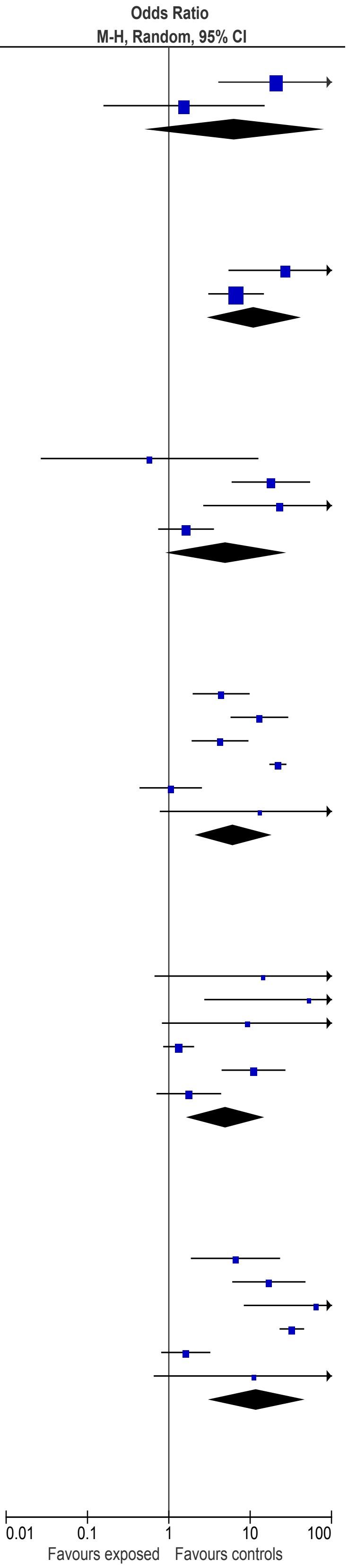

Figure S14: Forest plots of individual and pooled effects on cesarean delivery by type of CUA.
