# Supplementary material for: Impact of congenital uterine anomalies on obstetric and perinatal outcomes: systematic review and meta-analysis
Source: Facts Views Vis Obgyn. 2024 Mar 28;16(1):9–22. doi: 10.52054/FVVO.16.1.004 (PMC11198883; doi:10.52054/FVVO.16.1.004)
Supplement: Figure S15 — Forest plot of individual and pooled effects on IUGR/SGA of all CUA (combined). [file FVVinObGyn-16-9-gs015.pdf]

IUGR/SGA (all CUA)

Study or Subgroup	Exposed Events	Exposed Total	Controls Events	Controls Total	Weight	Odds Ratio M-H, Random, 95% CI	Year
Leible 1998	11	14	2	30	3.5%	51.33 [7.52, 350.26]	1998
Zlopasa 2007	25	105	8	182	9.5%	6.80 [2.94, 15.73]	2007
Hua 2011	26	203	5607	66753	13.7%	1.60 [1.06, 2.42]	2011
Takami 2014	13	80	568	5763	11.8%	1.77 [0.97, 3.23]	2014
Hiersch 2016	30	243	33	486	12.7%	1.93 [1.15, 3.25]	2016
Cahen-Peretz 2017	94	1251	12268	255048	15.3%	1.61 [1.30, 1.99]	2017
Mastrolia 2017	25	444	5720	279622		Not estimable	2017
Mastrolia 2018	78	1099	5720	279622	15.2%	3.66 [2.90, 4.61]	2018
Lü 2021	3	44	13	367	6.1%	1.99 [0.55, 7.28]	2021
Qiu 2022	15	127	138	1354	12.2%	1.18 [0.67, 2.08]	2022
**Total (95% CI)**		**3166**		**609605**	**100.0%**	**2.38 [1.59, 3.57]**	
Total events	295		24357				

Heterogeneity: Tau² = 0.27; Chi² = 52.28, df = 8 (P < 0.00001); I² = 85%

Test for overall effect: Z = 4.20 (P < 0.0001)

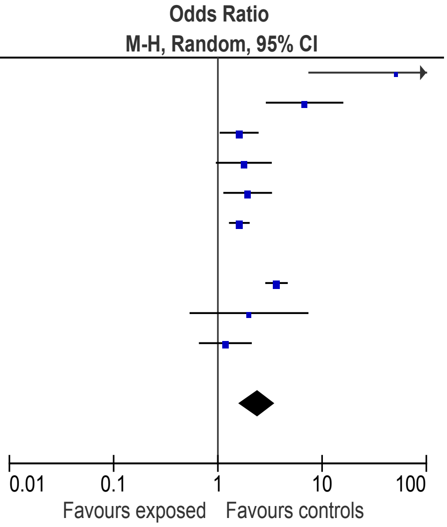

Figure S15: Forest plot of individual and pooled effects on IUGR/SGA of all CUA (combined).
