# Supplementary material for: Impact of congenital uterine anomalies on obstetric and perinatal outcomes: systematic review and meta-analysis
Source: Facts Views Vis Obgyn. 2024 Mar 28;16(1):9–22. doi: 10.52054/FVVO.16.1.004 (PMC11198883; doi:10.52054/FVVO.16.1.004)
Supplement: Figure S16 — Forest plots of individual and pooled effects on IUGR/SGA by type of CUA. [file FVVinObGyn-16-9-gs016.pdf]

IUGR/SGA by type of CUA

Study or Subgroup	Exposed Events	Exposed Total	Controls Events	Controls Total	Weight	Odds Ratio M-H, Random, 95% CI	Year
**2.13.1 Arcuate uterus**							
Zlopasa 2007	6	9	8	182	54.9%	43.50 [9.17, 206.31]	2007
Takami 2014	0	4	568	5763	45.1%	1.02 [0.05, 18.88]	2014
**Subtotal (95% CI)**		**13**		**5945**	**100.0%**	**7.99 [0.16, 405.90]**	
Total events	6		576				
Heterogeneity: Tau² = 6.68; Chi² = 5.68, df = 1 (P = 0.02); I² = 82%							
Test for overall effect: Z = 1.04 (P = 0.30)							
**2.13.2 Subseptate uterus**							
Zlopasa 2007	1	11	8	182	14.9%	2.17 [0.25, 19.13]	2007
Takami 2014	6	27	568	5763	85.1%	2.61 [1.05, 6.50]	2014
**Subtotal (95% CI)**		**38**		**5945**	**100.0%**	**2.54 [1.10, 5.89]**	
Total events	7		576				
Heterogeneity: Tau² = 0.00; Chi² = 0.02, df = 1 (P = 0.88); I² = 0%							
Test for overall effect: Z = 2.18 (P = 0.03)							
**2.13.3 Septate uterus**							
Zlopasa 2007	4	20	8	182	37.1%	5.44 [1.47, 20.05]	2007
Takami 2014	0	6	568	5763	16.3%	0.70 [0.04, 12.50]	2014
Qiu 2022	7	66	93	809	46.5%	0.91 [0.41, 2.06]	2022
**Subtotal (95% CI)**		**92**		**6754**	**100.0%**	**1.70 [0.43, 6.78]**	
Total events	11		669				
Heterogeneity: Tau² = 0.90; Chi² = 5.56, df = 2 (P = 0.06); I² = 64%							
Test for overall effect: Z = 0.75 (P = 0.45)							
**2.13.4 Bicornuate uterus**							
Zlopasa 2007	7	38	8	132	9.6%	3.50 [1.18, 10.39]	2007
Takami 2014	5	25	568	5763	11.8%	2.29 [0.85, 6.12]	2014
Mastrolia 2017	25	444	5720	279662	69.7%	2.86 [1.91, 4.28]	2017
Qiu 2022	4	25	32	372	8.9%	2.02 [0.65, 6.26]	2022
**Subtotal (95% CI)**		**532**		**285929**	**100.0%**	**2.75 [1.96, 3.86]**	
Total events	41		6328				
Heterogeneity: Tau² = 0.00; Chi² = 0.64, df = 3 (P = 0.89); I² = 0%							
Test for overall effect: Z = 5.88 (P < 0.00001)							
**2.13.5 Unicornuate uterus**							
Zlopasa 2007	2	4	8	182	19.3%	21.75 [2.71, 174.82]	2007
Takami 2014	0	3	568	5763	11.3%	1.31 [0.07, 25.31]	2014
Lü 2021	3	44	13	367	33.3%	1.99 [0.55, 7.28]	2021
Qiu 2022	4	36	13	173	36.1%	1.54 [0.47, 5.02]	2022
**Subtotal (95% CI)**		**87**		**6485**	**100.0%**	**2.74 [0.91, 8.29]**	
Total events	9		602				
Heterogeneity: Tau² = 0.52; Chi² = 5.17, df = 3 (P = 0.16); I² = 42%							
Test for overall effect: Z = 1.79 (P = 0.07)							
**2.13.6 Didelphys uterus**							
Zlopasa 2007	6	23	8	182	24.2%	7.68 [2.38, 24.73]	2007
Takami 2014	2	15	568	5763	16.8%	1.41 [0.32, 6.25]	2014
Cahen-Peretz 2017	18	258	4931	255048	59.0%	3.80 [2.35, 6.15]	2017
**Subtotal (95% CI)**		**296**		**260993**	**100.0%**	**3.82 [1.93, 7.56]**	
Total events	26		5507				
Heterogeneity: Tau² = 0.15; Chi² = 3.11, df = 2 (P = 0.21); I² = 36%							
Test for overall effect: Z = 3.84 (P = 0.0001)							

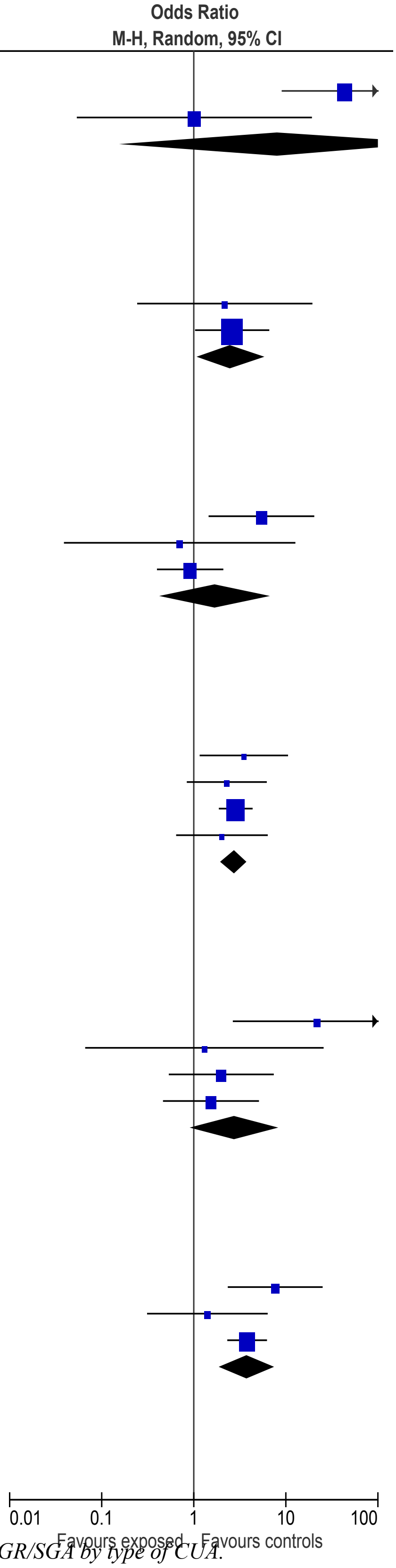

Figure S16: Forest plots of individual and pooled effects on IUGR/SGA by type of CUA.
