# Supplementary material for: Impact of congenital uterine anomalies on obstetric and perinatal outcomes: systematic review and meta-analysis
Source: Facts Views Vis Obgyn. 2024 Mar 28;16(1):9–22. doi: 10.52054/FVVO.16.1.004 (PMC11198883; doi:10.52054/FVVO.16.1.004)
Supplement: Figure S17 — Forest plot of individual and pooled effects on fetal mortality of all CUA (combined). [file FVVinObGyn-16-9-gs017.pdf]

Fetal mortality (all CUA)

Study or Subgroup	Exposed Events	Exposed Total	Controls Events	Controls Total	Weight	Odds Ratio M-H, Fixed, 95% CI	Year
Ben-Rafael 1991	9	245	2	413	2.7%	7.84 [1.68, 36.57]	1991
Leible 1998	2	15	0	30	0.5%	11.30 [0.51, 251.56]	1998
Zlopasa 2007	10	163	6	305	7.3%	3.26 [1.16, 9.13]	2007
Saravelos 2010	0	251	7	630	8.0%	0.17 [0.01, 2.90]	2010
Hua 2011	3	203	667	66753	7.5%	1.49 [0.47, 4.66]	2011
Li 2017	13	234	25	950	17.5%	2.18 [1.10, 4.32]	2017
Cahen-Peretz 2017	14	1251	1479	255048	26.7%	1.94 [1.14, 3.29]	2017
Mastrolia 2017	10	444	1903	279662		Not estimable	2017
Mastrolia 2018	14	1099	1903	279622	27.5%	1.88 [1.11, 3.20]	2018
Cai 2021	0	51	2	159	2.3%	0.61 [0.03, 12.95]	2021
**Total (95% CI)**		**3512**		**603910**	**100.0%**	**2.07 [1.56, 2.73]**	
Total events	65		4091				

Heterogeneity: Chi² = 8.88, df = 8 (P = 0.35); I² = 10%

Test for overall effect: Z = 5.07 (P < 0.00001)

Odds Ratio

M-H, Fixed, 95% CI

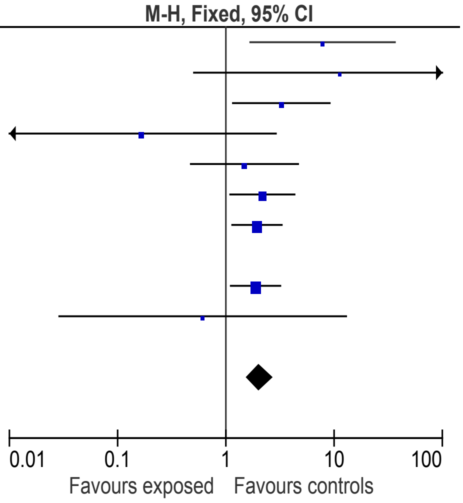

Figure S17: Forest plot of individual and pooled effects on fetal mortality of all CUA (combined).
