# Supplementary material for: Impact of congenital uterine anomalies on obstetric and perinatal outcomes: systematic review and meta-analysis
Source: Facts Views Vis Obgyn. 2024 Mar 28;16(1):9–22. doi: 10.52054/FVVO.16.1.004 (PMC11198883; doi:10.52054/FVVO.16.1.004)
Supplement: Figure S18 — Forest plots of individual and pooled effects on fetal mortality by type of CUA. [file FVVinObGyn-16-9-gs018.pdf]

Fetal mortality by type of CUA

Study or Subgroup	Exposed Events	Exposed Total	Controls Events	Controls Total	Weight	Odds Ratio M-H, Random, 95% CI	Year
**2.14.1 Arcuate uterus**							
Zlopasa 2007	0	10	6	305	48.8%	2.19 [0.12, 41.57]	2007
Saravelos 2010	0	101	7	630	51.2%	0.41 [0.02, 7.23]	2010
**Subtotal (95% CI)**		**111**		**935**	**100.0%**	**0.93 [0.12, 7.25]**	
Total events	0		13				
Heterogeneity: Tau^2 = 0.00; Chi^2 = 0.70, df = 1 (P = 0.40); I^2 = 0%							
Test for overall effect: Z = 0.07 (P = 0.94)							
**2.14.2 Subseptate uterus**							
Zlopasa 2007	1	17	6	305	100.0%	3.11 [0.35, 27.44]	2007
**Subtotal (95% CI)**		**17**		**305**	**100.0%**	**3.11 [0.35, 27.44]**	
Total events	1		6				
Heterogeneity: Not applicable							
Test for overall effect: Z = 1.02 (P = 0.31)							
**2.14.3 Septate uterus**							
Zlopasa 2007	1	31	6	305	64.1%	1.66 [0.19, 14.26]	2007
Saravelos 2010	0	106	7	630	35.9%	0.39 [0.02, 6.88]	2010
**Subtotal (95% CI)**		**137**		**935**	**100.0%**	**0.99 [0.18, 5.52]**	
Total events	1		13				
Heterogeneity: Tau^2 = 0.00; Chi^2 = 0.68, df = 1 (P = 0.41); I^2 = 0%							
Test for overall effect: Z = 0.01 (P = 0.99)							
**2.14.4 Bicornuate uterus**							
Zlopasa 2007	5	60	6	305	20.2%	4.53 [1.34, 15.36]	2007
Saravelos 2010	0	29	7	630	3.6%	1.41 [0.08, 25.26]	2010
Mastrolia 2017	10	444	1903	279662	76.2%	3.36 [1.79, 6.31]	2017
**Subtotal (95% CI)**		**533**		**280597**	**100.0%**	**3.46 [2.00, 5.99]**	
Total events	15		1916				
Heterogeneity: Tau^2 = 0.00; Chi^2 = 0.57, df = 2 (P = 0.75); I^2 = 0%							
Test for overall effect: Z = 4.44 (P < 0.00001)							
**2.14.5 Unicornuate uterus**							
Zlopasa 2007	0	6	6	305	4.8%	3.54 [0.18, 69.75]	2007
Saravelos 2010	0	5	7	630	4.8%	7.56 [0.38, 149.28]	2010
Li 2017	13	234	25	950	90.4%	2.18 [1.10, 4.32]	2017
**Subtotal (95% CI)**		**245**		**1885**	**100.0%**	**2.36 [1.23, 4.54]**	
Total events	13		38				
Heterogeneity: Tau^2 = 0.00; Chi^2 = 0.72, df = 2 (P = 0.70); I^2 = 0%							
Test for overall effect: Z = 2.59 (P = 0.010)							
**2.14.6 Didelphys uterus**							
Zlopasa 2007	3	39	6	305	25.8%	4.15 [1.00, 17.33]	2007
Saravelos 2010	0	10	7	630	6.1%	3.96 [0.21, 73.91]	2010
Cai 2021	9	79	14	251	68.1%	2.18 [0.90, 5.24]	2021
**Subtotal (95% CI)**		**128**		**1186**	**100.0%**	**2.67 [1.29, 5.51]**	
Total events	12		27				
Heterogeneity: Tau^2 = 0.00; Chi^2 = 0.65, df = 2 (P = 0.72); I^2 = 0%							
Test for overall effect: Z = 2.65 (P = 0.008)							

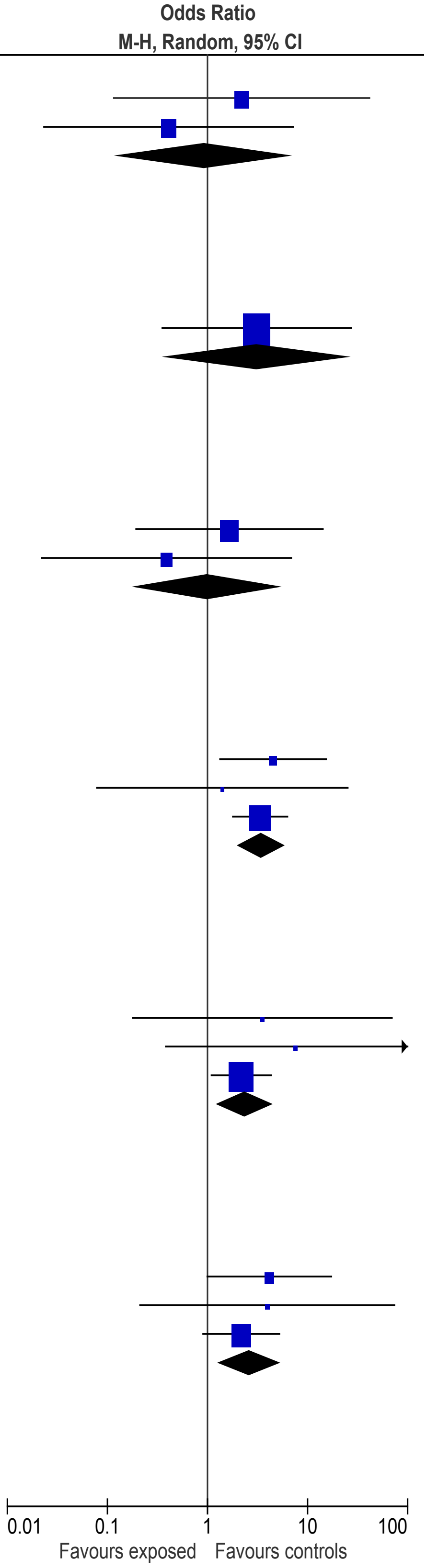

Figure S18: Forest plots of individual and pooled effects on fetal mortality by type of CUA.
