# Supplementary material for: Impact of congenital uterine anomalies on obstetric and perinatal outcomes: systematic review and meta-analysis
Source: Facts Views Vis Obgyn. 2024 Mar 28;16(1):9–22. doi: 10.52054/FVVO.16.1.004 (PMC11198883; doi:10.52054/FVVO.16.1.004)
Supplement: Figure S19 — Forest plot of individual and pooled effects on perinatal mortality of all CUA (combined). [file FVVinObGyn-16-9-gs019.pdf]

Perinatal mortality (all CUA)

Study or Subgroup	Exposed Events	Exposed Total	Controls Events	Controls Total	Weight	Odds Ratio M-H, Random, 95% CI	Year
Crane 1992	2	52	1	121	3.7%	4.80 [0.43, 54.14]	1992
Zlopasa 2007	16	163	8	305	16.7%	4.04 [1.69, 9.66]	2007
Mastrolia 2017	11	444	2085	279662	23.1%	3.38 [1.86, 6.16]	2017
Cahen-Peretz 2017	21	1251	2264	255048	27.7%	1.91 [1.24, 2.94]	2017
Li 2017	21	234	31	950	23.8%	2.92 [1.65, 5.19]	2017
Ouyang 2020	21	206	1	314	5.1%	35.53 [4.74, 266.31]	2020
**Total (95% CI)**		**2350**		**536400**	**100.0%**	**3.28 [2.01, 5.36]**	
Total events	92		4390				

Heterogeneity: Tau² = 0.18; Chi² = 11.31, df = 5 (P = 0.05); I² = 56%
Test for overall effect: Z = 4.74 (P < 0.00001)

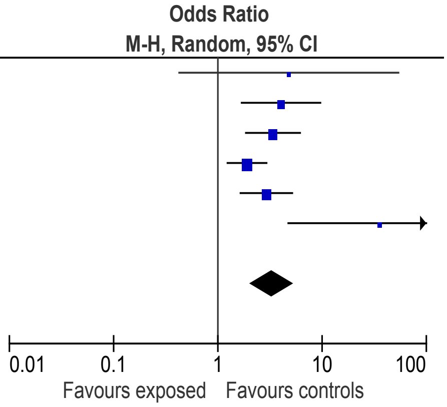

Figure S19: Forest plot of individual and pooled effects on perinatal mortality of all CUA (combined).
