# Supplementary material for: Impact of congenital uterine anomalies on obstetric and perinatal outcomes: systematic review and meta-analysis
Source: Facts Views Vis Obgyn. 2024 Mar 28;16(1):9–22. doi: 10.52054/FVVO.16.1.004 (PMC11198883; doi:10.52054/FVVO.16.1.004)
Supplement: Figure S20 — Forest plots of individual and pooled effects on perinatal mortality by type of CUA. [file FVVinObGyn-16-9-gs020.pdf]

Perinatal mortality by type of CUA

Study or Subgroup	Exposed Events	Exposed Total	Controls Events	Controls Total	Weight	Odds Ratio M-H, Random, 95% CI
**2.15.1 Arcuate uterus**						
Zlopasa 2007	1	10	8	305	100.0%	4.13 [0.47, 36.57]
**Subtotal (95% CI)**		**10**		**305**	**100.0%**	**4.13 [0.47, 36.57]**
Total events	1		8			
Heterogeneity: Not applicable						
Test for overall effect: Z = 1.27 (P = 0.20)						
**2.15.2 Subseptate uterus**						
Zlopasa 2007	2	17	8	305	100.0%	4.95 [0.97, 25.37]
**Subtotal (95% CI)**		**17**		**305**	**100.0%**	**4.95 [0.97, 25.37]**
Total events	2		8			
Heterogeneity: Not applicable						
Test for overall effect: Z = 1.92 (P = 0.06)						
**2.15.3 Septate uterus**						
Crane 1992	0	2	1	121	17.7%	16.07 [0.52, 499.52]
Zlopasa 2007	2	31	8	305	82.3%	2.56 [0.52, 12.63]
**Subtotal (95% CI)**		**33**		**426**	**100.0%**	**3.55 [0.83, 15.08]**
Total events	2		9			
Heterogeneity: Tau² = 0.00; Chi² = 0.93, df = 1 (P = 0.33); I² = 0%						
Test for overall effect: Z = 1.71 (P = 0.09)						
**2.15.4 Bicornuate uterus**						
Crane 1992	0	35	1	121	9.7%	1.13 [0.05, 28.39]
Zlopasa 2007	7	60	8	305	90.3%	4.90 [1.71, 14.09]
**Subtotal (95% CI)**		**95**		**426**	**100.0%**	**4.25 [1.56, 11.60]**
Total events	7		9			
Heterogeneity: Tau² = 0.00; Chi² = 0.73, df = 1 (P = 0.39); I² = 0%						
Test for overall effect: Z = 2.83 (P = 0.005)						
**2.15.5 Didelphys uterus**						
Crane 1992	2	13	1	121	27.8%	21.82 [1.83, 260.17]
Zlopasa 2007	4	39	8	305	72.2%	4.24 [1.22, 14.81]
**Subtotal (95% CI)**		**52**		**426**	**100.0%**	**6.69 [1.59, 28.15]**
Total events	6		9			
Heterogeneity: Tau² = 0.34; Chi² = 1.34, df = 1 (P = 0.25); I² = 25%						
Test for overall effect: Z = 2.59 (P = 0.010)						
**2.15.6 Unicornuate uterus**						
Crane 1992	0	2	1	121	2.6%	16.07 [0.52, 499.52]
Li 2017	21	234	31	950	93.9%	2.92 [1.65, 5.19]
Zlopasa 2007	0	6	8	305	3.5%	2.69 [0.14, 51.76]
**Subtotal (95% CI)**		**242**		**1376**	**100.0%**	**3.05 [1.75, 5.31]**
Total events	21		40			
Heterogeneity: Tau² = 0.00; Chi² = 0.93, df = 2 (P = 0.63); I² = 0%						
Test for overall effect: Z = 3.93 (P < 0.0001)						

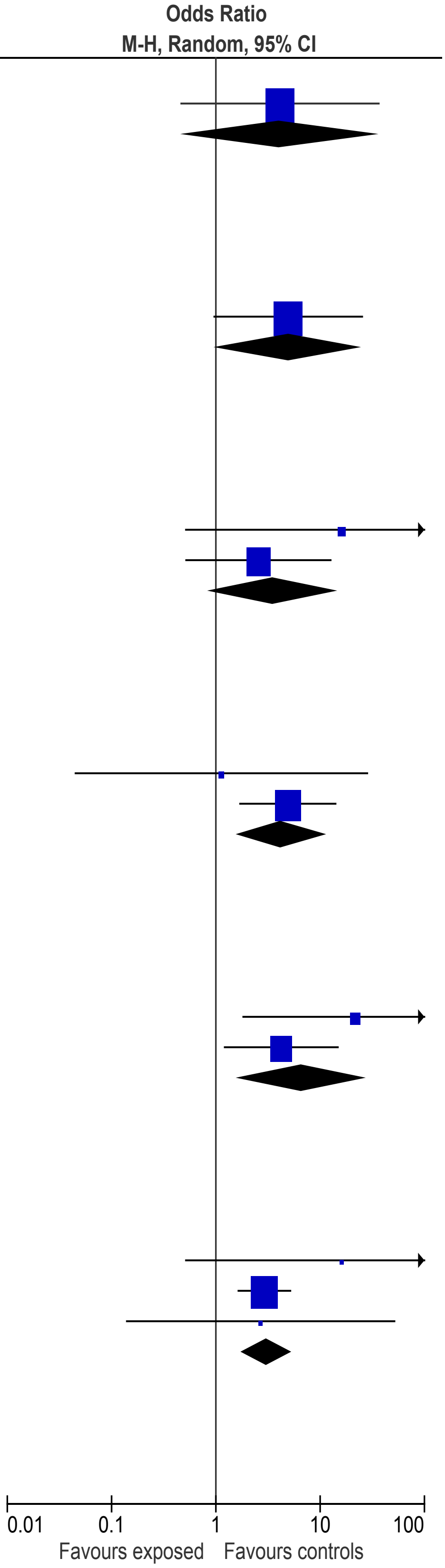

Figure S20: Forest plots of individual and pooled effects on perinatal mortality by type of CUA.
