# Supplementary material for: Impact of congenital uterine anomalies on obstetric and perinatal outcomes: systematic review and meta-analysis
Source: Facts Views Vis Obgyn. 2024 Mar 28;16(1):9–22. doi: 10.52054/FVVO.16.1.004 (PMC11198883; doi:10.52054/FVVO.16.1.004)
Supplement: Supplementary file 24 [file FVVinObGyn-16-9-a001.pdf]

Appendix 1: NLM (PUBMED) structured search

(congenital uterine anomalies OR müllerian anomalies OR arcuate uterus OR septate uterus OR subseptate uterus OR bicornuate uterus OR unicornuate uterus OR didelphis uterus OR dysmorphic uterus OR T-shaped uterus) AND (‘pregnancy OR miscarriage OR spontaneous abortion OR ectopic pregnancy OR delivery OR live birth’, ‘pregnancy outcome OR perinatal outcome OR premature birth OR prematurity OR preterm delivery OR preterm labor OR premature rupture of membranes OR preterm premature rupture of membranes OR intrauterine growth restriction OR (infant, small for gestational age) OR placental abruption OR cesarean delivery OR fetal malposition OR abnormal presentation OR fetal mortality OR stillbirth OR perinatal mortality OR pregnancy complications OR obstetric labor complications AND (1000/1/1:2021/10/3[pdat])) Filters: **from 1000/1/1 - 2022/04/30[pdat])**

((((“congenital”[MeSH Subheading] OR “congenital”[All Fields] OR “congenitally”[All Fields]) AND (“uterine anomalies”[Supplementary Concept] OR “uterine anomalies”[All Fields] OR “uterine anomalies”[All Fields])) OR (“mullerian”[All Fields] AND (“abnormalities”[MeSH Subheading] OR “abnormalities”[All Fields] OR “anomalies”[All Fields] OR “anomalie”[All Fields] OR “anomaly”[All Fields])) OR (“arcuate”[All Fields] AND (“uterus”[MeSH Terms] OR “uterus”[All Fields] OR “uteri”[All Fields])) OR ((“septate”[All Fields] OR “septated”[All Fields] OR “septates”[All Fields] OR “septation”[All Fields] OR “septations”[All Fields]) AND (“uterus”[MeSH Terms] OR “uterus”[All Fields] OR “uteri”[All Fields])) OR (“subseptate”[All Fields] AND (“uterus”[MeSH Terms] OR “uterus”[All Fields] OR “uteri”[All Fields])) OR (“bicornuate”[All Fields] AND (“uterus”[MeSH Terms] OR “uterus”[All Fields] OR “uteri”[All Fields])) OR (“unicornuate”[All Fields] AND (“uterus”[MeSH Terms] OR “uterus”[All Fields] OR “uteri”[All Fields])) OR ((“didelphis”[MeSH Terms] OR “didelphis”[All Fields]) AND (“uterus”[MeSH Terms] OR “uterus”[All Fields] OR “uteri”[All Fields])) OR ((“congenital abnormalities”[MeSH Terms] OR (“congenital”[All Fields] AND “abnormalities”[All Fields]) OR “congenital abnormalities”[All Fields] OR “dysmorphism”[All Fields] OR “dysmorphisms”[All Fields] OR “dysmorphic”[All Fields]) AND (“uterus”[MeSH Terms] OR “uterus”[All Fields] OR “uteri”[All Fields])) OR (“T-shaped”[All Fields] AND (“uterus”[MeSH Terms] OR “uterus”[All Fields] OR “uteri”[All Fields]))) AND ((“pregnancy”[MeSH Terms] OR “pregnancy”[All Fields] OR “pregnancies”[All Fields] OR “pregnancy s”[All Fields] OR (“abortion, spontaneous”[MeSH Terms] OR (“abortion”[All Fields] AND “spontaneous”[All Fields]) OR “spontaneous abortion”[All Fields] OR “miscarriage”[All Fields] OR “miscarriages”[All Fields]) OR (“abortion, spontaneous”[MeSH Terms] OR (“abortion”[All Fields] AND “spontaneous”[All Fields]) OR “spontaneous abortion”[All Fields] OR (“spontaneous”[All Fields] AND “abortion”[All Fields])) OR (“pregnancy, ectopic”[MeSH Terms] OR (“pregnancy”[All Fields] AND “ectopic”[All Fields]) OR “ectopic pregnancy”[All Fields] OR (“ectopic”[All Fields] AND “pregnancy”[All Fields])) OR (“deliveries”[All Fields] OR “delivery, obstetric”[MeSH Terms] OR (“delivery”[All Fields] AND “obstetric”[All Fields]) OR “obstetric delivery”[All Fields] OR “delivery”[All Fields]) OR ((“live birth”[MeSH Terms] OR (“live”[All Fields] AND “birth”[All Fields]) OR “live birth”[All Fields]) AND (“pregnancy outcome”[MeSH Terms] OR (“pregnancy”[All Fields] AND “outcome”[All Fields]) OR “pregnancy outcome”[All Fields])) OR ((“perinatal”[All Fields] OR “perinatally”[All Fields] OR “perinatals”[All Fields]) AND (“outcome”[All Fields] OR “outcomes”[All Fields])) OR (“premature birth”[MeSH Terms] OR (“premature”[All Fields] AND “birth”[All Fields]) OR “premature birth”[All Fields]) OR (“premature birth”[MeSH Terms] OR (“premature”[All Fields] AND “birth”[All Fields]) OR “premature birth”[All Fields] OR “premature”[All Fields] OR “prematurely”[All Fields] OR “prematures”[All Fields] OR “prematurities”[All Fields] OR “prematurity”[All Fields]) OR (“premature birth”[MeSH Terms] OR (“premature”[All Fields] AND “birth”[All Fields]) OR “premature birth”[All Fields] OR (“preterm”[All Fields] AND “delivery”[All Fields]) OR “preterm delivery”[All Fields]) OR (“obstetric labor, premature”[MeSH Terms] OR (“obstetric”[All Fields] AND “labor”[All Fields] AND “premature”[All Fields]) OR “premature obstetric labor”[All Fields] OR (“preterm”[All Fields] AND “labor”[All Fields]) OR “preterm labor”[All Fields]) OR (“fetal membranes, premature rupture”[MeSH Terms] OR (“fetal”[All Fields] AND “membranes”[All Fields] AND

"premature"[All Fields] AND "rupture"[All Fields]) OR "premature rupture fetal membranes"[All Fields] OR ("premature"[All Fields] AND "rupture"[All Fields] AND "membranes"[All Fields]) OR "premature rupture of membranes"[All Fields]) OR (("premature birth"[MeSH Terms] OR ("premature"[All Fields] AND "birth"[All Fields]) OR "premature birth"[All Fields] OR "preterm"[All Fields] OR "preterms"[All Fields]) AND ("fetal membranes, premature rupture"[MeSH Terms] OR ("fetal"[All Fields] AND "membranes"[All Fields] AND "premature"[All Fields] AND "rupture"[All Fields]) OR "premature rupture fetal membranes"[All Fields] OR ("premature"[All Fields] AND "rupture"[All Fields] AND "membranes"[All Fields]) OR "premature rupture of membranes"[All Fields])) OR ("fetal growth retardation"[MeSH Terms] OR ("fetal"[All Fields] AND "growth"[All Fields] AND "retardation"[All Fields]) OR "fetal growth retardation"[All Fields] OR ("intrauterine"[All Fields] AND "growth"[All Fields] AND "restriction"[All Fields]) OR "intrauterine growth restriction"[All Fields]) OR ("infant, small for gestational age"[MeSH Terms] OR ("infant"[All Fields] AND "small"[All Fields] AND "gestational"[All Fields] AND "age"[All Fields]) OR "small for gestational age infant"[All Fields] OR ("infant"[All Fields] AND "small"[All Fields] AND "gestational"[All Fields] AND "age"[All Fields]) OR "infant small for gestational age"[All Fields]) OR ("abruptio placentae"[MeSH Terms] OR ("abruptio"[All Fields] AND "placentae"[All Fields]) OR "abruptio placentae"[All Fields] OR ("placental"[All Fields] AND "abruption"[All Fields]) OR "placental abruption"[All Fields]) OR ("cesarean section"[MeSH Terms] OR ("cesarean"[All Fields] AND "section"[All Fields]) OR "cesarean section"[All Fields] OR ("cesarean"[All Fields] AND "delivery"[All Fields]) OR "cesarean delivery"[All Fields]) OR (("fetale"[All Fields] OR "fetally"[All Fields] OR "fetals"[All Fields] OR "fetus"[MeSH Terms] OR "fetus"[All Fields] OR "fetal"[All Fields] OR "foetal"[All Fields]) AND ("malposition"[All Fields] OR "malpositioned"[All Fields] OR "malpositioning"[All Fields] OR "malpositionings"[All Fields] OR "malpositions"[All Fields])) OR (("abnormal"[All Fields] OR "abnormalities"[MeSH Subheading] OR "abnormalities"[All Fields] OR "congenital abnormalities"[MeSH Terms] OR ("congenital"[All Fields] AND "abnormalities"[All Fields]) OR "congenital abnormalities"[All Fields] OR "abnormality"[All Fields] OR "abnormally"[All Fields] OR "abnormals"[All Fields] OR "abnormities"[All Fields] OR "abnormity"[All Fields]) AND ("present"[All Fields] OR "presentation"[All Fields] OR "presentations"[All Fields] OR "presented"[All Fields] OR "presenter"[All Fields] OR "presenter s"[All Fields] OR "presenters"[All Fields] OR "presenting"[All Fields] OR "presents"[All Fields])) OR ("fetal mortality"[MeSH Terms] OR ("fetal"[All Fields] AND "mortality"[All Fields]) OR "fetal mortality"[All Fields]) OR ("stillbirth"[MeSH Terms] OR "stillbirth"[All Fields] OR "stillbirths"[All Fields]) OR ("perinatal mortality"[MeSH Terms] OR ("perinatal"[All Fields] AND "mortality"[All Fields]) OR "perinatal mortality"[All Fields] OR "perinatal death"[MeSH Terms] OR ("perinatal"[All Fields] AND "death"[All Fields]) OR "perinatal death"[All Fields] OR ("perinatal"[All Fields] AND "mortality"[All Fields])) OR ("pregnancy complications"[MeSH Terms] OR ("pregnancy"[All Fields] AND "complications"[All Fields]) OR "pregnancy complications"[All Fields]) OR ("obstetric labor complications"[MeSH Terms] OR ("obstetric"[All Fields] AND "labor"[All Fields] AND "complications"[All Fields]) OR "obstetric labor complications"[All Fields])) AND 1000/01/01:2021/10/03[Date - Publication])) AND (1000/1/1:2022/04/30[pdat])

Translations

congenital: "congenital"[Subheading] OR "congenital"[All Fields] OR "congenitally"[All Fields]

uterine anomalies: "Uterine Anomalies"[Supplementary Concept] OR "Uterine Anomalies"[All Fields] OR "uterine anomalies"[All Fields]

anomalies: "abnormalities"[Subheading] OR "abnormalities"[All Fields] OR "anomalies"[All Fields] OR "anomalie"[All Fields] OR "anomaly"[All Fields]

uterus: "uterus"[MeSH Terms] OR "uterus"[All Fields] OR "uteri"[All Fields]

septate: "septate"[All Fields] OR "septated"[All Fields] OR "septates"[All Fields] OR "septation"[All Fields] OR "septations"[All Fields]

uterus: "uterus"[MeSH Terms] OR "uterus"[All Fields] OR "uteri"[All Fields]

uterus: "uterus"[MeSH Terms] OR "uterus"[All Fields] OR "uteri"[All Fields]

uterus: "uterus"[MeSH Terms] OR "uterus"[All Fields] OR "uteri"[All Fields]

uterus: "uterus"[MeSH Terms] OR "uterus"[All Fields] OR "uteri"[All Fields]

didelphis: "didelphis"[MeSH Terms] OR "didelphis"[All Fields]

uterus: "uterus"[MeSH Terms] OR "uterus"[All Fields] OR "uteri"[All Fields]

dysmorphic: “congenital abnormalities”[MeSH Terms] OR (“congenital”[All Fields] AND “abnormalities”[All Fields]) OR “congenital abnormalities”[All Fields] OR “dysmorphism”[All Fields] OR “dysmorphisms”[All Fields] OR “dysmorphic”[All Fields]

uterus: “uterus”[MeSH Terms] OR “uterus”[All Fields] OR “uteri”[All Fields]

uterus: “uterus”[MeSH Terms] OR “uterus”[All Fields] OR “uteri”[All Fields]

‘pregnancy: “pregnancy”[MeSH Terms] OR “pregnancy”[All Fields] OR “pregnancies”[All Fields] OR “pregnancy’s”[All Fields]

miscarriage: “abortion, spontaneous”[MeSH Terms] OR (“abortion”[All Fields] AND “spontaneous”[All Fields]) OR “spontaneous abortion”[All Fields] OR “miscarriage”[All Fields] OR “miscarriages”[All Fields]

spontaneous abortion: “abortion, spontaneous”[MeSH Terms] OR (“abortion”[All Fields] AND “spontaneous”[All Fields]) OR “spontaneous abortion”[All Fields] OR (“spontaneous”[All Fields] AND “abortion”[All Fields])

ectopic pregnancy: “pregnancy, ectopic”[MeSH Terms] OR (“pregnancy”[All Fields] AND “ectopic”[All Fields]) OR “ectopic pregnancy”[All Fields] OR (“ectopic”[All Fields] AND “pregnancy”[All Fields])

delivery: “deliveries”[All Fields] OR “delivery, obstetric”[MeSH Terms] OR (“delivery”[All Fields] AND “obstetric”[All Fields]) OR “obstetric delivery”[All Fields] OR “delivery”[All Fields]

live birth’,: “live birth”[MeSH Terms] OR (“live”[All Fields] AND “birth”[All Fields]) OR “live birth”[All Fields]

‘pregnancy outcome: “pregnancy outcome”[MeSH Terms] OR (“pregnancy”[All Fields] AND “outcome”[All Fields]) OR “pregnancy outcome”[All Fields]

perinatal: “perinatal”[All Fields] OR “perinatally”[All Fields] OR “perinatals”[All Fields]

outcome: “outcome”[All Fields] OR “outcomes”[All Fields]

premature birth: “premature birth”[MeSH Terms] OR (“premature”[All Fields] AND “birth”[All Fields]) OR “premature birth”[All Fields]

prematurity: “premature birth”[MeSH Terms] OR (“premature”[All Fields] AND “birth”[All Fields]) OR “premature birth”[All Fields] OR “premature”[All Fields] OR “prematurely”[All Fields] OR “prematures”[All Fields] OR “prematurities”[All Fields] OR “prematurity”[All Fields]

preterm delivery: “premature birth”[MeSH Terms] OR (“premature”[All Fields] AND “birth”[All Fields]) OR “premature birth”[All Fields] OR (“preterm”[All Fields] AND “delivery”[All Fields]) OR “preterm delivery”[All Fields]

preterm labor: “obstetric labor, premature”[MeSH Terms] OR (“obstetric”[All Fields] AND “labor”[All Fields] AND “premature”[All Fields]) OR “premature obstetric labor”[All Fields] OR (“preterm”[All Fields] AND “labor”[All Fields]) OR “preterm labor”[All Fields]

premature rupture of membranes: “fetal membranes, premature rupture”[MeSH Terms] OR (“fetal”[All Fields] AND “membranes”[All Fields] AND “premature”[All Fields] AND “rupture”[All Fields]) OR “premature rupture fetal membranes”[All Fields] OR (“premature”[All Fields] AND “rupture”[All Fields] AND “membranes”[All Fields]) OR “premature rupture of membranes”[All Fields]

preterm: “premature birth”[MeSH Terms] OR (“premature”[All Fields] AND “birth”[All Fields]) OR “premature birth”[All Fields] OR “preterm”[All Fields] OR “preterms”[All Fields]

premature rupture of membranes: “fetal membranes, premature rupture”[MeSH Terms] OR (“fetal”[All Fields] AND “membranes”[All Fields] AND “premature”[All Fields] AND “rupture”[All Fields]) OR “premature rupture fetal membranes”[All Fields] OR (“premature”[All Fields] AND “rupture”[All Fields] AND “membranes”[All Fields]) OR “premature rupture of membranes”[All Fields]

intrauterine growth restriction: “fetal growth retardation”[MeSH Terms] OR (“fetal”[All Fields] AND “growth”[All Fields] AND “retardation”[All Fields]) OR “fetal growth retardation”[All Fields] OR (“intrauterine”[All Fields] AND “growth”[All Fields] AND “restriction”[All Fields]) OR “intrauterine growth restriction”[All Fields]

infant, small for gestational age: “infant, small for gestational age”[MeSH Terms] OR (“infant”[All Fields] AND “small”[All Fields] AND “gestational”[All Fields] AND “age”[All Fields]) OR “small for gestational age infant”[All Fields] OR (“infant”[All Fields] AND “small”[All Fields] AND “gestational”[All Fields] AND “age”[All Fields]) OR “infant small for gestational age”[All Fields]

placental abruption: “abruptio placentae”[MeSH Terms] OR (“abruptio”[All Fields] AND “placentae”[All Fields]) OR “abruptio placentae”[All Fields] OR (“placental”[All Fields] AND “abruption”[All Fields]) OR “placental abruption”[All Fields]

cesarean delivery: “cesarean section”[MeSH Terms] OR (“cesarean”[All Fields] AND

"section"[All Fields]) OR "cesarean section"[All Fields] OR ("cesarean"[All Fields] AND "delivery"[All Fields]) OR "cesarean delivery"[All Fields]

fetal: "fetale"[All Fields] OR "fetally"[All Fields] OR "fetals"[All Fields] OR "fetus"[MeSH Terms] OR "fetus"[All Fields] OR "fetal"[All Fields] OR "foetal"[All Fields]

malposition: "malposition"[All Fields] OR "malpositioned"[All Fields] OR "malpositioning"[All Fields] OR "malpositionings"[All Fields] OR "malpositions"[All Fields]

abnormal: "abnormal"[All Fields] OR "abnormalities"[Subheading] OR "abnormalities"[All Fields] OR "congenital abnormalities"[MeSH Terms] OR ("congenital"[All Fields] AND "abnormalities"[All Fields]) OR "congenital abnormalities"[All Fields] OR "abnormality"[All Fields] OR "abnormally"[All Fields] OR "abnormals"[All Fields] OR "abnormities"[All Fields] OR "abnormity"[All Fields]

presentation: "present"[All Fields] OR "presentation"[All Fields] OR "presentations"[All Fields] OR "presented"[All Fields] OR "presenter"[All Fields] OR "presenter's"[All Fields] OR "presenters"[All Fields] OR "presenting"[All Fields] OR "presents"[All Fields]

fetal mortality: "fetal mortality"[MeSH Terms] OR ("fetal"[All Fields] AND "mortality"[All Fields]) OR "fetal mortality"[All Fields]

stillbirth: "stillbirth"[MeSH Terms] OR "stillbirth"[All Fields] OR "stillbirths"[All Fields]

perinatal mortality: "perinatal mortality"[MeSH Terms] OR ("perinatal"[All Fields] AND "mortality"[All Fields]) OR "perinatal mortality"[All Fields] OR "perinatal death"[MeSH Terms] OR ("perinatal"[All Fields] AND "death"[All Fields]) OR "perinatal death"[All Fields] OR ("perinatal"[All Fields] AND "mortality"[All Fields])

pregnancy complications: "pregnancy complications"[MeSH Terms] OR ("pregnancy"[All Fields] AND "complications"[All Fields]) OR "pregnancy complications"[All Fields]

obstetric labor complications: "obstetric labor complications"[MeSH Terms] OR ("obstetric"[All Fields] AND "labor"[All Fields] AND "complications"[All Fields]) OR "obstetric labor complications"[All Fields]
