# Supplementary material for: Impact of congenital uterine anomalies on obstetric and perinatal outcomes: systematic review and meta-analysis
Source: Facts Views Vis Obgyn. 2024 Mar 28;16(1):9–22. doi: 10.52054/FVVO.16.1.004 (PMC11198883; doi:10.52054/FVVO.16.1.004)
Supplement: Supplementary file 25 [file FVVinObGyn-16-9-a002.pdf]

Appendix 2: INCLUDED STUDIES: Newcastle-Ottawa Scale scores

First author, year	Newcastle-Ottawa Scale domains								Total Score	Quality (AHRQ standards)†
	Selection*				Comparability*	Outcome*				
	Representativeness of the exposed cohort	Selection of the non-exposed cohort	Ascertainment of exposure	Demonstration that outcome of interest was not present at start of study	Comparability of cohorts on the basis of the design or analysis controlled for confounders	Assessment of outcome	Follow-up long enough for outcomes to occur	Adequacy of follow-up of cohorts		
Ben-Rafael, 1991	*	*	*		*	*	*	*	7	Good
Cooney, 1998	*	*		*	*	*	*	*	7	Good
Leible, 1998			*	*	*	*	*	*	6	Fair
Erez, 2007	*	*		*	*	*	*	*	7	Good
Zlopasa, 2007	*	*	*	*	*	*	*	*	8	Good
Ban-Frangez, 2009	*	*	*	*	*	*	*	*	8	Good
Sugiura-Ogasawara, 2010	*	*		*	**	*	*	*	8	Good
Saravelos, 2010	*	*	*	*	**	*	*	*	9	Good
Tomazevic, 2010	*	*	*	*	**	*	*	*	9	Good
Hua, 2011	*	*	*	*	**	*	*	*	9	Good
Jayaprakasan, 2011	*	*	*	*	*	*	*	*	8	Good
Crane, 2012	*	*		*	*	*	*	*	7	Good
Takami, 2014	*	*	*	*	*	*	*	*	8	Good
Hiersch, 2016	*	*		*	**	*	*	*	8	Good
Li, 2017	*	*		*	*	*	*	*	7	Good
Ozgur, 2017	*	*		*	*	*	*	*	7	Good
Mastrolia, 2017	*	*		*	*	*	*	*	7	Good
Cahen-Peretz, 2017	*	*		*	**	*	*	*	9	Good
Mastrolia, 2018	*	*		*	*	*	*	*	7	Good
Ples, 2018	*	*	*	*	**	*	*	*	9	Good
Prior, 2018	*	*	*	*	**	*	*	*	9	Good
Surrey, 2018	*	*	*	*	**	*	*	*	9	Good
Chen, 2018a	*	*		*	*	*	*	*	7	Good
Chen, 2018b	*	*		*	*	*	*	*	7	Good
Ouyang, 2020	*	*	*	*	**	*	*	*	9	Good

Cai, 2021	*	*	*	*	*	*	*	*	8	Good
Kong, 2021	*	*	*	*	**	*	*	*	9	Good
Lü, 2021	*	*	*	*	**	*	*	*	9	Good
Zambrotta, 2021	*	*	*	*	**	*	*	*	9	Good
Zhang, 2021	*	*		*	**	*	*	*	8	Good
Marianna, 2022	*	*	*	*	**	*	*	*	9	Good
Qiu, 2022	*	*	*	*	**	*	*	*	9	Good

*** Specific scoring criteria:**

– Representativeness of the exposed cohort: Hospital-based cohorts including all exposed patients (n≥30) registered during the observation period were granted with one star.
– Selection of the non-exposed cohort: Hospital-bases cohorts including all non-exposed patients (n≥30) from the same population registered during the observation period were granted with one star.
– Ascertainment of exposure: müllerian abnormality assessment by laparoscopy, ultrasonography or histeroscopy was granted with one star.
– Comparability of cohorts on the basis of the design or analysis controlled for confounders:
    - Studies controlling basic potential confounders (age, parity and number of transferred embryos in IVF patients) were granted with one star.
    - Studies controlling also one or more additional relevant potential confounders were granted with two stars.
– Assessment of outcome: confirmation of outcome provided by clinical records was granted with one star.
– Adequacy of follow-up of cohorts: Follow up rate > 90% was granted with one star.

[†]**USA Agency for Healthcare Research and Quality standards:**

– Good quality: 3 or 4 stars in selection domain AND 1 or 2 stars in comparability domain AND 2 or 3 stars in outcome/exposure domain
– Fair quality: 2 stars in selection domain AND 1 or 2 stars in comparability domain AND 2 or 3 stars in outcome/exposure domain
– Poor quality: 0 or 1 star in selection domain OR 0 stars in comparability domain OR 0 or 1 stars in outcome/exposure domain
